# Reduction-cleavable desferrioxamine B pulldown system enriches Ni(ii)-superoxide dismutase from a *Streptomyces* proteome†

**DOI:** 10.1039/d3cb00097d

**Published:** 2023-10-02

**Authors:** Jenny Ni, James L. Wood, Melanie Y. White, Norbert Lihi, Todd E. Markham, Joseph Wang, Peter T. Chivers, Rachel Codd

**Affiliations:** a School of Medical Sciences, The University of Sydney New South Wales 2006 Australia rachel.codd@sydney.edu.au; b Charles Perkins Centre, The University of Sydney New South Wales 2006 Australia; c ELKH-DE Mechanisms of Complex Homogeneous and Heterogeneous Chemical Reactions Research Group, Department of Inorganic and Analytical Chemistry, University of Debrecen Debrecen H-4032 Hungary; d Department of Chemistry, Durham University Durham DH1 3LE UK; e Department of Biosciences, Durham University Durham DH1 3LE UK

## Abstract

Two resins with the hydroxamic acid siderophore desferrioxamine B (DFOB) immobilised as a free ligand or its Fe(iii) complex were prepared to screen the *Streptomyces pilosus* proteome for proteins involved in siderophore-mediated Fe(iii) uptake. The resin design included a disulfide bond to enable the release of bound proteins under mild reducing conditions. Proteomics analysis of the bound fractions did not identify proteins associated with siderophore-mediated Fe(iii) uptake, but identified nickel superoxide dismutase (NiSOD), which was enriched on the apo-DFOB-resin but not the Fe(iii)-DFOB-resin or the control resin. While DFOB is unable to sequester Fe(iii) from sites deeply buried in metalloproteins, the coordinatively unsaturated Ni(ii) ion in NiSOD is present in a surface-exposed loop region at the N-terminus, which might enable partial chelation. The results were consistent with the notion that the apo-DFOB-resin formed a ternary complex with NiSOD, which was not possible for either the coordinatively saturated Fe(iii)-DFOB-resin or the non-coordinating control resin systems. In support, ESI-TOF-MS measurements from a solution of a model Ni(ii)-SOD peptide and DFOB showed signals that correlated with a ternary Ni(ii)-SOD peptide–DFOB complex. Although any biological implications of a DFOB–NiSOD complex are unclear, the work shows that the metal coordination properties of siderophores might influence an array of metal-dependent biological processes beyond those established in iron uptake.

## Abstract 2

Two resins with the hydroxamic acid siderophore desferrioxamine B (DFOB) immobilised as a free ligand or its Fe(iii) complex prepared to screen the *Streptomyces pilosus* proteome for proteins involved in siderophore-mediated Fe(iii) uptake enriched for Ni(ii)-superoxide dismutase on the apo-DFOB resin.

## Graphical abstract

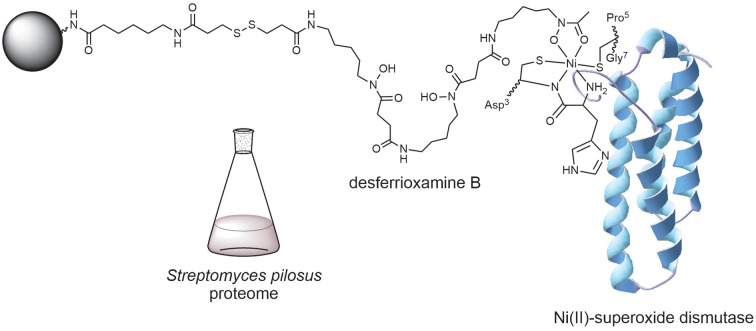

## Introduction

The production of siderophores, which are low-molecular-weight organic compounds with high Fe(iii) affinity, is a widespread strategy used by environmental and pathogenic bacteria and fungi to acquire essential iron.^1,2^ Siderophores contain different types of functional groups (hydroxamic acid, catechol, α-hydroxycarboxylic acid, thiazoli(di)ne, *N*-nitroso-*N*-hydroxylamine) to sequester and bind Fe(iii) sourced from the local terrestrial or marine environment, or from transferrin or lactoferrin in the mammalian host to establish an infection.^3^ The Fe(iii)–siderophore complex is recognised by cell-surface proteins, which initiates a protein-mediated import cascade that provides the Fe(iii)–siderophore complex to the cytoplasm.

The essentiality of iron and the multiple proteins and entry points in siderophore-mediated Fe(iii) uptake identifies this pathway as an attractive antibacterial target.^4–7^ Small-molecule inhibitors of proteins in this pathway could attenuate bacterial iron supply and reduce fitness. A selection of siderophore binding proteins and Fe(iii)–siderophore outer membrane transporter proteins from Gram-positive and Gram-negative bacteria, respectively, have been characterised by molecular methods and X-ray crystallography.^8–11^ Developing methods to identify the complement of native proteins involved in the Fe(iii)–siderophore mediated uptake pathway would be useful.^12^ It remains possible that this targeted approach could identify proteins that recognise apo- or Fe(iii)–siderophores but have no known roles in iron uptake.

**Scheme 1:**
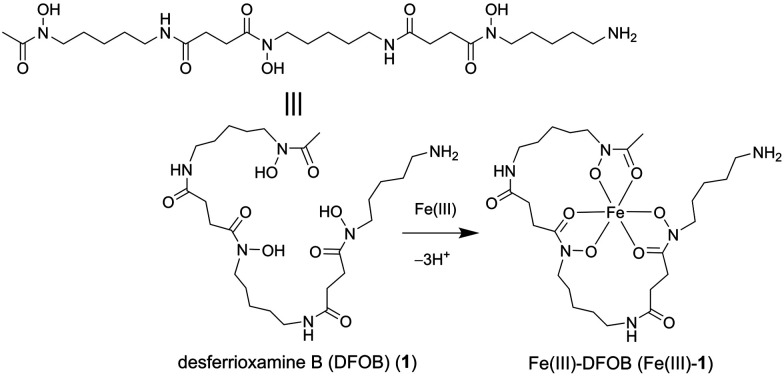
Desferrioxamine B as a free ligand (DFOB, 1) or an Fe(iii) complex (Fe(iii)-1).

This work set to design an affinity matrix using an immobilised siderophore to select for binding proteins from the native proteome of the cognate siderophore-producing species. The archetypal hydroxamic acid siderophore desferrioxamine B (DFOB, Scheme 1, **1**) was selected for this study since 1 has an established and robust semi-synthetic chemistry^13^ and is produced in measurable quantities in cultures of *Streptomyces pilosus* and other actinomycetes as a correlate of the production of attendant proteins.^14,15^

The work aimed to develop a method to access native proteins and/or protein–protein complexes that might remain bound to the siderophore probe upon elution. This led to introducing a reduction-cleavable disulfide bond in 1 in a region not involved in Fe(iii) coordination to enable elution using a mild reducing agent, such as dithiothreitol or tris(2-carboxyethyl)phosphine (TCEP).

The work describes the production of a reduction-cleavable resin containing 1 and its use in a pulldown assay against the *S. pilosus* proteome. Instead of proteins involved in siderophore-mediated Fe(iii) uptake, the work identified Ni(ii) superoxide dismutase (NiSOD) as a binding partner of the 1-displaying resin, which together with results from the Fe(iii)-1 and control systems, was consistent with the notion of the formation of a ternary complex between the 1-displaying resin and NiSOD, made possible due to the accessibility of the tetracoordinate Ni(ii) ion in a loop region at the NiSOD periphery. The proposed formation of a 1-NiSOD complex, which was indirectly corroborated from experiments using a model Ni(ii)-SOD peptide system, reflects the broader metal chelating capacity of siderophores, which could have implications for the myriad environmental and physiological metal-dependent biological processes.

## Results and discussion

### Design and preparation of thiol-containing probes

**Scheme 2:**
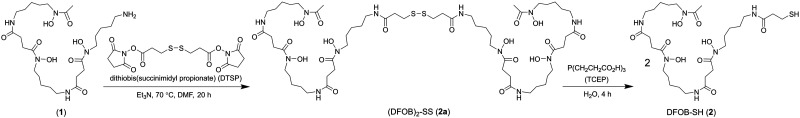
Synthesis from DFOB (1) the DFOB-containing disulfide precursor (DFOB)_2_-SS (2a) and DFOB-SH (2).

A previous analogue of 1 named DFOB-(SS)_1_[001] with a disulfide motif embedded in the terminal diamine-containing region^16^ was first considered as a starting compound to generate the target resin. The low yield of DFOB-(SS)_1_[001], as produced from *S. pilosus* cultures in cystamine-supplemented medium, prompted a different approach. Rather than embedding the disulfide unit within 1, a thiol motif was installed at the terminal amine group. Thiol-containing *N*-(DFOB)-3-mercaptopropanamide (DFOB-SH (2)) was prepared from the TCEP-mediated reduction of the homodimeric precursor dithiobis(*N*-DFOB-propanamide) ((DFOB)_2_-SS (2a)), which itself was prepared from the reaction between 1 and dithiobis(succinimidyl propionate) (DTSP) (Scheme 2).

**Scheme 3:**
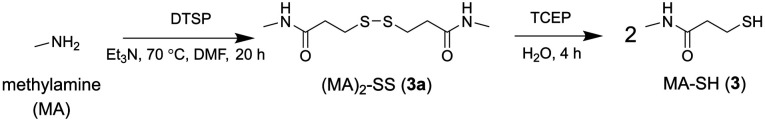
Synthesis from methylamine (MA) the MA-containing disulfide precursor (MA)_2_-SS (3a) and MA-SH (3).

A control probe was prepared to assess non-selective binding using non-coordinating methylamine (MA) to replace 1. The same protocol for the synthesis of 2a and 2 was used to generate the dimeric precursor (MA)_2_-SS (3a) with TCEP-mediated reduction generating MA-SH (3) (Scheme 3).

The dimeric precursors 2a and 3a were characterised in solution using LC-MS prior to reduction to generate 2 or 3, respectively, which were purified using immobilised metal affinity chromatography (IMAC)^17^ or flash chromatography (ESI† Fig. S11 and S12). The formation of Fe(iii)-2 was confirmed from LC-MS and electronic absorption spectroscopy measurements (ESI† Fig. S13 and S14). Both probes were prepared at increased scale, HPLC purified and characterised by HRMS and/or NMR spectroscopy (ESI† Fig. S1–S6 (2) and Fig S7–S11 (3/3a)).

### Preparation and characterisation of resins displaying 2 or 3

**Scheme 4:**
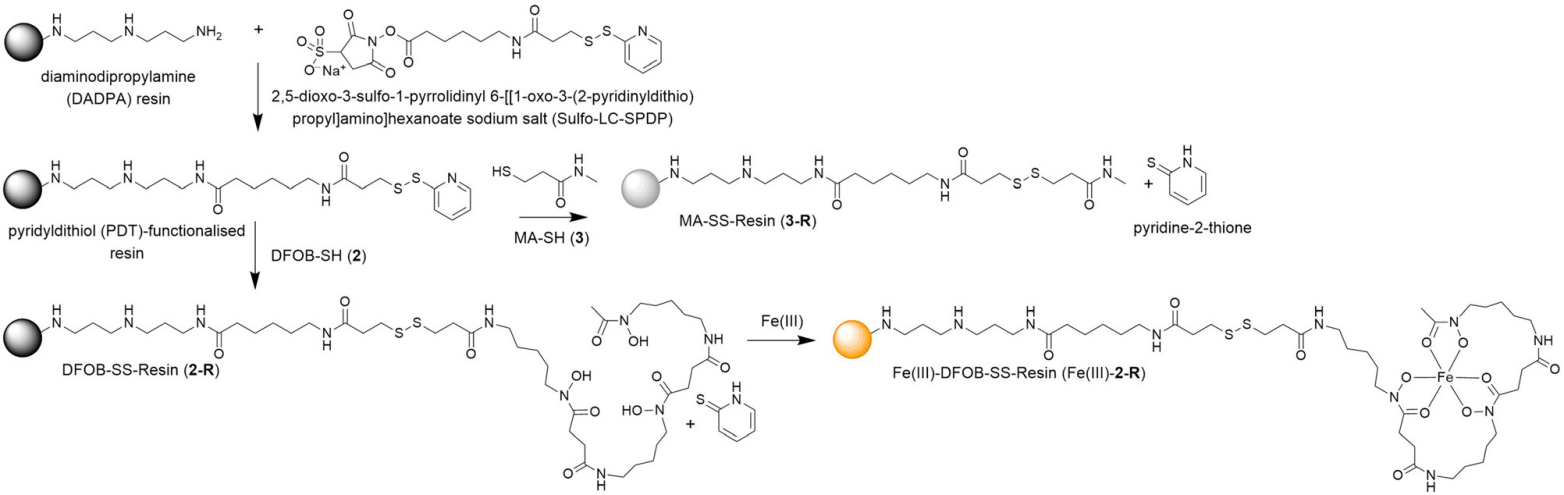
Preparation of reduction-cleavable resins: DFOB-SS-Resin (2-R), Fe(iii)-DFOB-SS-Resin (Fe(iii)-2-R), or MA-SS-Resin (3-R).

A pyridyldithiol (PDT)-functionalised resin freshly prepared from a reaction between a diaminodipropylamine (DADPA)-bearing resin and Sulfo-LC-SPDP^18^ was incubated with DFOB-SH (2) or MA-SH (3) to give DFOB-SS-Resin (2-R) or MA-SS-Resin (3-R), respectively. The disulfide-exchange reactions were monitored by the formation of pyridine-2-thione (*λ*_max_ = 343 nm) (Scheme 4). Some siderophore binding proteins are reported to recognise the siderophore in both its apo and holo (Fe(iii)-bound) form,^19,20^ which warranted the study of 2-R and Fe(iii)-2-R. A solution of Fe(iii) was adsorbed onto 2-R and the excess Fe(iii) removed to give Fe(iii)-DFOB-SS-Resin (Fe(iii)-2-R).

**Fig. 1:**
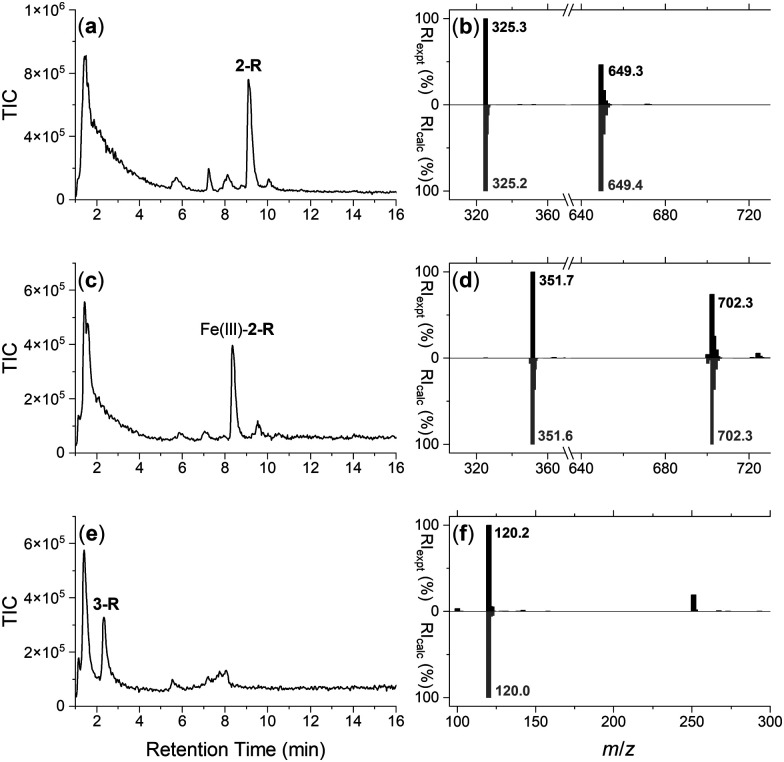
LC-MS traces reported as total ion current (TIC) from the eluent from a TCEP wash of the (a) DFOB-SS-Resin (2-R), (c) Fe(iii)-DFOB-SS-Resin (Fe(iii)-2-R), or (e) MA-SS-Resin (3-R), with experimental (black) MS signals from the peak at (a) 9.1 min, (c) 8.3 min, or (e) 2.4 min in (b), (d) or (f), respectively, with calculated signals ([M + 2H]^2+^ and/or [M + H]^+^ adducts) for DFOB-SH (2), Fe(iii)-DFOB-SH (Fe(iii)-2) or MA-SH (3) in grey.

The integrity of the 2-R-, Fe(iii)-2-R-, and 3-R resins was confirmed by washing each resin with TCEP and identifying by LC-MS the respective probes 2, Fe(iii)-2 or 3, in the eluent (Fig. 1). The signal at 1–2 min in each trace was due to TCEP, with well resolved signals at 9.1 min (Fig. 1a), 8.3 min (Fig. 1c) or 2.4 min (Fig. 1e) corresponding with 2, Fe(iii)-2 or 3, respectively. The experimental MS signal from each peak corresponded with the calculated values for adducts of the relevant species (Fig. 1b, d and f) with the isotope pattern characteristic of Fe(iii) species evident in the signal for Fe(iii)-2. The signal at *m*/*z* 251 in the eluent from 3-R was due to TCEP ([M + H]^+^*m*/*z*_calc_ = 251.1).

### Proteome production, pull-down, and proteomics analysis

**Fig. 2:**
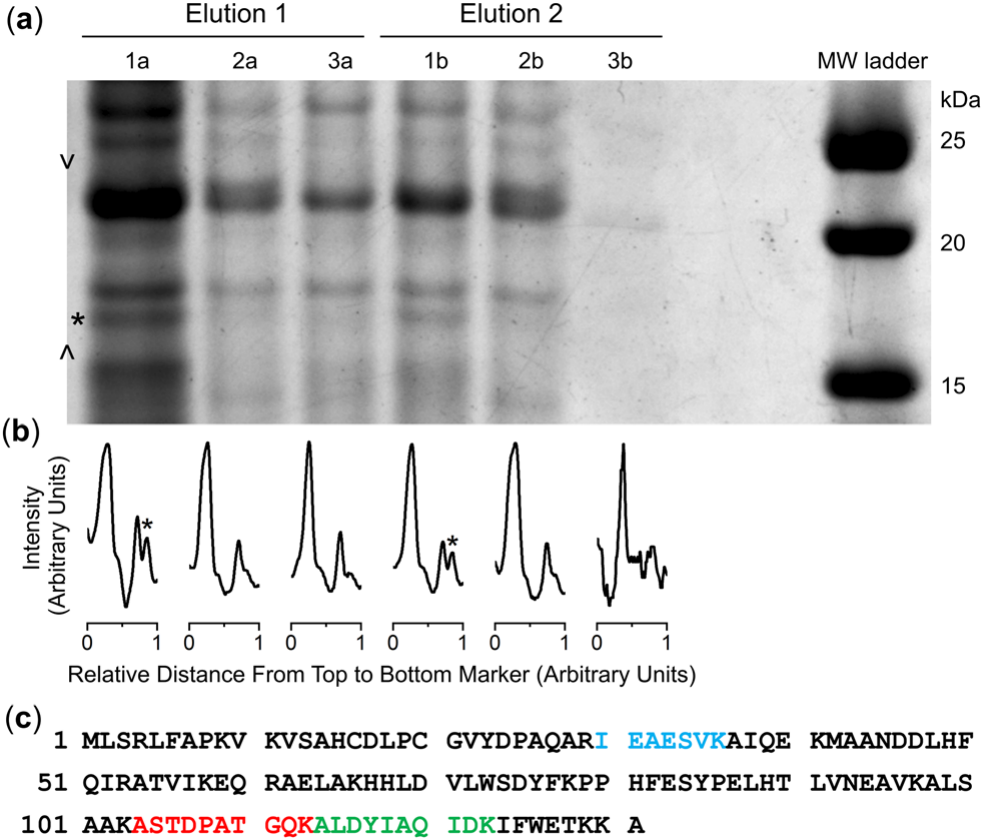
SDS-polyacrylamide gel electrophoresis (a) of fractions eluted from the DFOB-SH-(2-R) (lanes 1a, 1b), Fe(iii)-DFOB- (Fe(iii)-2-R) (lanes 2a, 2b), or MA- (3-R) containing resin (lanes 3a, 3b) in two TCEP elution fractions, with lane-matched densitometry analysis (b) for the marked region (> <), and (c) peptide fragments identified (band *) as matched to NiSOD (Accession code: P80734).

Cultures of the native DFOB-producer *S. pilosus* were grown in iron-depleted medium to upregulate the biosynthesis of 1 and the cognate proteins involved in Fe(iii)-1 import. Sub-samples were withdrawn from the cultures at 2-d internals up to the 10-d harvest date and analysed for Fe(iii)-coordinating species using an Fe(iii) addition assay as an indirect measure of 1 production (Fig. S16, ESI†). The proteome was generated from the cell pellet by ultrasonic processing in the presence of an EDTA-free solution of protease inhibitors and the cell debris removed by centrifugation. An aliquot of the *S. pilosus* proteome was incubated with 2-R, Fe(iii)-2-R or 3-R, and the unbound proteins removed by washing the resin bed until no protein was detected by the Bradford assay. A solution of TCEP was applied to the resin, with these fractions testing positive for protein (Fig. S17, ESI†). A sample of the proteome and fractions collected from the binding-wash-elution process from 2-R, Fe(iii)-2-R or 3-R were analysed by SDS-PAGE (Fig. S18, ESI†), with an excerpt of a region of interest (Fig. 2a) further analysed using densitometry (Fig. 2b).

The protein profile eluted from the 3-R control resin (Fig. 2, lane 3a) was broadly similar with the profile from the 2-R resin (lane 1a) and the Fe(iii)-2-R resin (lane 2a), showing a degree of non-specific binding. This reflected a shortcoming of using disulfide-containing probes in proteome screening.^21,22^ Proteins containing surface-exposed Cys residues could displace both the target and control probes with equal effect from the resin, which would result in non-specific binding.

Despite this, several regions of interest were identified in the gel showing differences in band intensities among the 2-R, Fe(iii)-2-R and 3-R systems. One band of interest appeared in the 2-R system (lane 1a, asterisked) that was weaker in both the Fe(iii)-2-R and 3-R systems. This band was evident in both the elution fractions (lane 1a, 1b) specific to the 2-R system, and was resolved by densitometry analysis (Fig. 2b and Fig. S19, ESI†).

### Identification of NiSOD and a putative enrichment mechanism

The protein band selectively enriched on the 2-R resin was cut out from the gel, digested and subject to proteomics analysis. The protein was identified as Ni(ii) superoxide dismutase (NiSOD) based on data from *Streptomyces seoulensis*, with 20% sequence coverage (Fig. 2c and Fig. S20, Table S1, ESI†).

NiSOD was first isolated and characterised from *Streptomyces* sp. IMSNU-1 and *S. coelicolor*,^23^ with NiSOD from *S. coelicolor* and *S. seoulensis* since structurally characterised by X-ray crystallography.^24,25^ NiSOD from *Streptomyces* sp. and other bacterial species contains two Cys residues (Cys2, Cys6) which are positioned in the N-terminal loop region and act as thiolate donors to the Ni(ii) ion.^26^ The square planar Ni(ii) geometry is completed by N donor atoms from His1 (N-terminal amine) and Cys2 (amide), with a square pyramidal Ni(iii) centre accessible upon coordination from the His1 imidazole (N^*δ*^) side chain.^24,25,27–33^

**Scheme 5:**
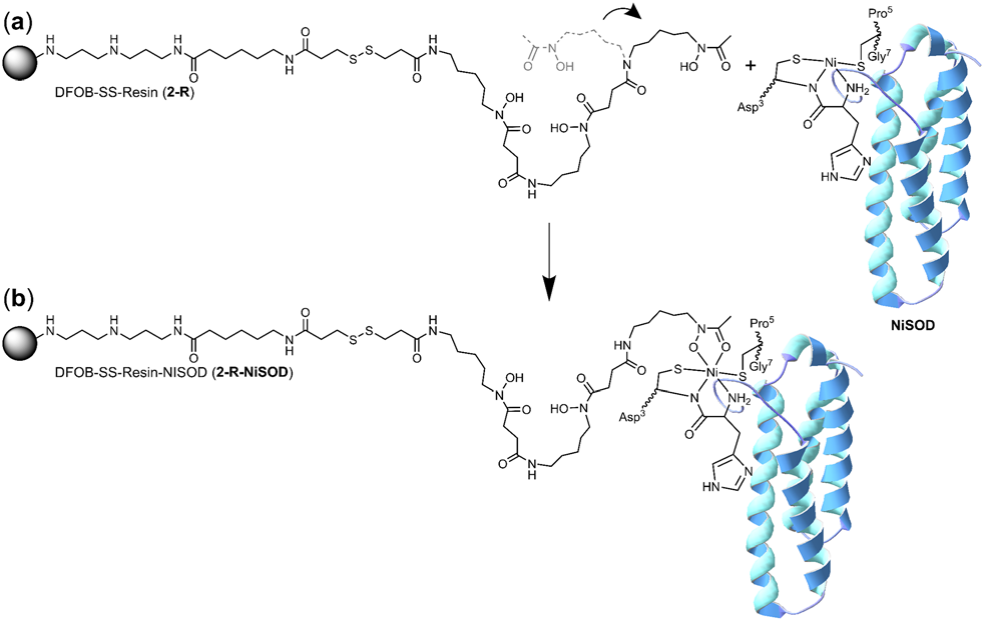
One hydroxamic acid unit from the apo-DFOB resin system (2-R) flexing (a) to bind the Ni(ii) ion present at the periphery of NiSOD to form a ternary complex (b).

It is conceivable that the enrichment of NiSOD on the 2-R resin occurred *via* the formation of a ternary apo-DFOB-Ni(ii)-SOD complex in which one of the bidentate *O*,*O*′-hydroxamic acid groups bound to and reorganised the native tetracoordinate Ni(ii) site to generate a hexacoordinate species (Scheme 5). This proposition was consistent with the observation that NiSOD was enriched only on the apo-DFOB-SS-Resin (2-R) in which the hydroxamic acid groups remained available for metal binding. In contrast, the coordinatively-saturated Fe(iii)-DFOB-SS-resin (Fe(iii)-2-R) system and the non-coordinating control MA-SS-Resin (3-R) systems would be unable to bind NiSOD, as consistent with the minimal enrichment of this band in these latter two systems. The molecular weight of the protein band was estimated at 15.7 kDa similar with NiSOD from *S. seoulensis* at 14.7 kDa (UniProt).

The proposed complexation between one hydroxamic acid unit of 1 in 2-R and the Ni(ii) ion in NiSOD is supported by known Ni(ii)-hydroxamic acid coordination chemistry,^34–41^ including complexes with mixed mercapto-hydroxamic acid ligands,^42^ and acetohydroxamic acid-based inhibition of the binuclear Ni(ii) site in urease.^43–45^ The stability constant of the Ni(ii)-1 (1-H_4_^+^) complex with mono-hydroxamate coordination ([Ni(ii)-1-H_−1_]) is log *K* = 4.36, with the di-([Ni(ii)-1-H_−2_]^−^) or tri-([Ni(ii)-1-H_−3_]^2−^) hydroxamate complexes having stability constants log *K* = 7.70 or log *K* = 10.90, respectively.^46^ The magnitude of these stability constants, particularly as compared with analogous Fe(iii) complexes (log *K* 21.84 ([Fe(iii)-1-H_−2_]^2+^); log *K* 30.60 ([Fe(iii)-1-H_−3_]^+^) correlate with the capacity of resin-bound 1 to contribute as a mono-hydroxamate bidentate chelate to the tetradentate NiSOD coordination sphere, without the Ni(ii) ion in NiSOD being transferred to the resin.

### NiSOD in *Streptomyces* species

Aside from the metal coordinating Cys residues, NiSOD from *Streptomyces* species contains no other Cys residues. This would preclude any non-specific binding effects resulting from Cys-mediated disulfide exchange. This supported the enrichment of NiSOD from *S. pilosus* on the 2-R resin system could result from a selective interaction between the protein and the 1 motif. NiSOD from other genera, including strains of *Prochlorococcus marinus*, *Synechococcus* sp. and *Trichodesmium erythraeum* contain four Cys residues, including the two Ni(ii)-coordinating residues and two residues towards the C-terminus.^24,25,47^ The NiSOD from these species may not have been enriched on the 2-R system due to non-specific disulfide exchange.

### Evaluating the viability of a ternary NiSOD-1 or -2 complex

**Fig. 3:**
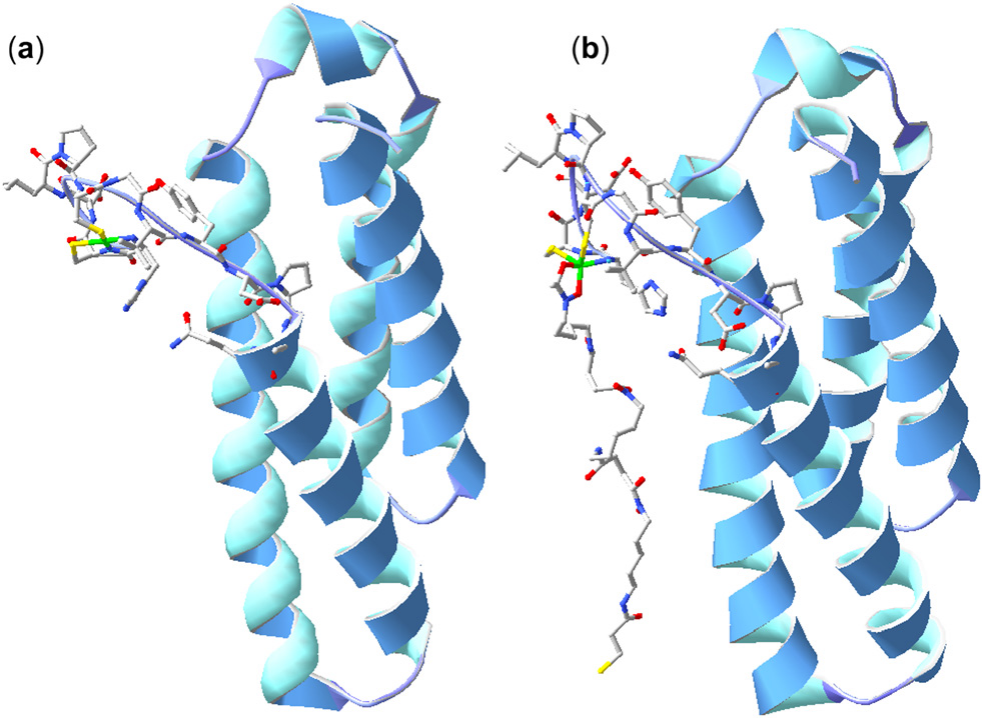
X-ray structure (H atoms omitted for clarity) of NiSOD from *S. coelicolor* (PDB: 1t6u) (a) and model of 2 bound to the Ni(ii) site *via* the *N*-acetyl-positioned hydroxamic acid unit (b).

Further studies were undertaken to evaluate the viability of forming a ternary NiSOD-1 (or -2) complex. An initial study to examine 1 and NiSOD interactions measured UV-Vis spectra from solutions of excess 1 (>1000×) with recombinant NiSOD to examine whether 1 could extract the Ni(ii) ion from NiSOD. This was found not possible, with no obvious spectral changes observed under these conditions. Spectra corrected for NiSOD showed minor changes that could be due to factors other than ternary complex formation (Fig. S21, ESI†). Molecular modelling and ESI-TOF-MS studies were next undertaken. The energy minimised model of NiSOD using start coordinates from X-ray data^24^ and 2 built as bound to the Ni(ii) site *via* the *N*-acetyl hydroxamate group gave a distorted Ni(ii) trigonal prismatic coordination geometry. The Ni(ii) ion and six atoms in the first coordination sphere showed good overlay (RMS 1.91 Å) with trigonal prismatic Ni(ii) complexes characterised by X-ray crystallography.^48^ The linear conformation of 2, as built into the starting coordinates, showed the potential for the formation of additional stabilising hydrogen bonding interactions between the amide and hydroxamic acid groups and the residue side chains (E17, N31, R39, R47) in the NiSOD backbone (Fig. 3). The model showed the displacement of the His1 residue upon mono-hydroxamate binding, with the mobility of this residue established as part of the NiSOD mechanism.^28,30,49–52^ The modelling was informative, but alone was insufficient to support the proposed ternary complex.

### ESI-TOF-MS measurements

To examine the formation of a ternary species between the apo-DFOB-resin and NiSOD, ESI-TOF-MS measurements were acquired from a model system. The Ni(ii) complex of a model NiSOD peptide HisCysAspLeuProCysGlyValTyr-NH_2_ was mixed with 1 at a peptide:1 ratio of 1 : 1 or 1 : 5 at pH 7.0 and introduced into the ESI† source. Earlier studies have showed this minimal peptide sequence models both the coordination mode and the catalytic features of the NiSOD enzyme.^32,53^

**Fig. 4:**
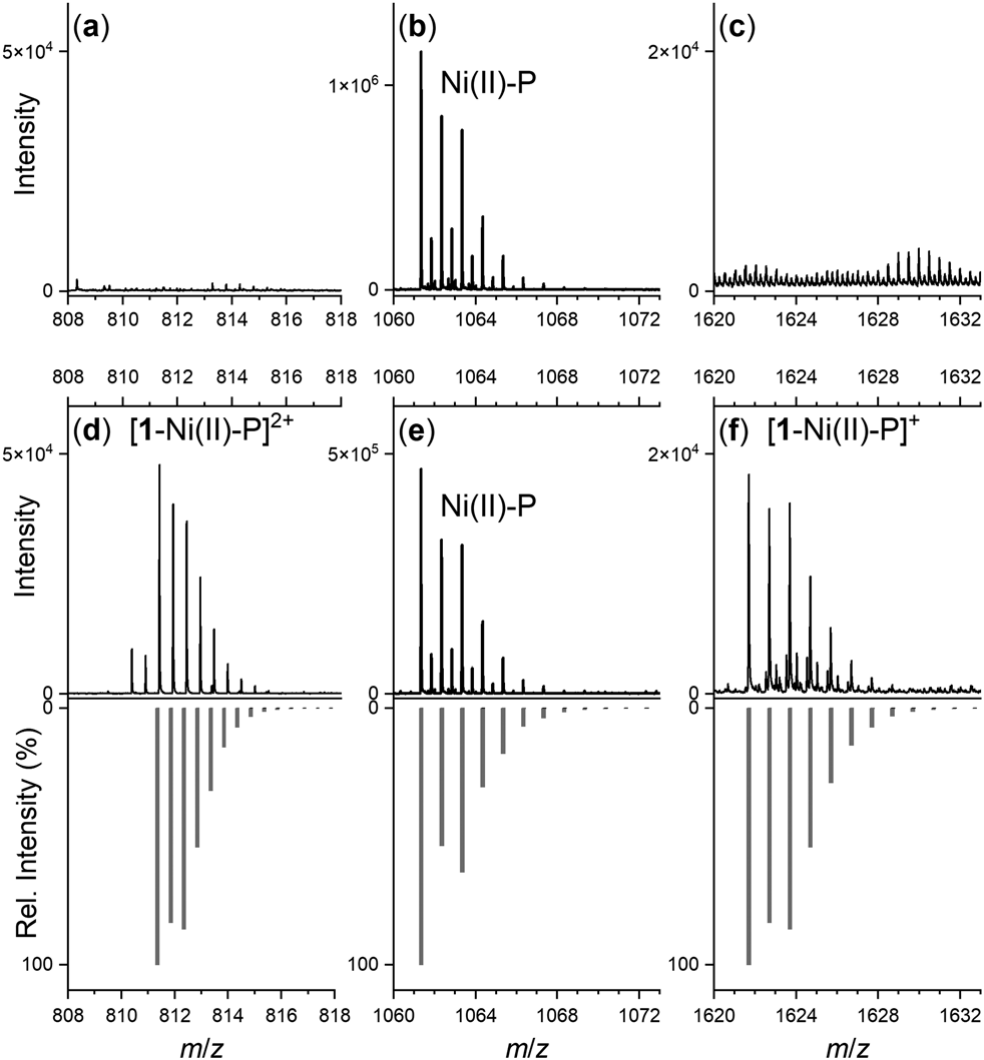
Regions of the ESI-TOF-MS data from solutions of the Ni(ii)-SOD peptide (Ni(ii)-P) in the absence (a)–(c) or presence (d)–(f) of 1 (peptide:1 = 1 : 5), showing experimental (black) or calculated (grey) signals ascribable to Ni(ii)-P (b) and (e) or 1-Ni(ii)-P adducts (d) and (f).

Spectra from the solution of the Ni(ii)-peptide alone showed signals ascribed to double protonated (Table 1) and single protonated adducts of the Ni(ii)-peptide (Fig. 4b). These signals were also present in solutions containing the Ni(ii)-peptide and 1 (Fig. 4e), with additional signals observed in this mixture that correlated with theoretical signals (grey) for the double protonated (*m*/*z* = 811.3480) (Fig. 4d) and single protonated (*m*/*z* = 1621.6944) (Fig. 4f) adducts of a 1 : 1 1 : Ni(ii)-peptide complex. The signal intensity for these species increased in a concentration-dependent manner (peptide:1 1 : 5 > 1 : 1) and provided evidence to support the proposed formation of a ternary Ni(ii)-SOD-1 complex.

**Table 1:** Species (*m*/*z* values (observed, calculated)) from ESI-TOF-MS experiments from a solution of a Ni(ii)-peptide model (L) of NiSOD and 1

Stoichiometry	*m*/*z* (obs)	*m*/*z* (calc)
1: H[C_25_H_48_N_6_O_8_]^+^	561.3598	561.3606
H_5_L: [C_43_H_65_N_12_O_12_S_2_]^+^	1005.4217	1005.4281
H_3_[NiH_–1_L]: H_4_[NiC_43_H_60_N_12_O_12_S_2_]^+^	1061.3411	1061.3478
H_4_[NiH_–1_L]: H_4_[NiC_43_H_60_N_12_O_12_S_2_]^2+^	531.1729	531.1775
H_4_[Ni(H_–1_L)(DFOB)]: H_4_[NiC_68_H_108_N_18_O_20_S_2_]^2+^	811.3480	811.3542
H_3_[Ni(H_–1_L)(DFOB)]: H_3_[NiC_68_H_108_N_18_O_20_S_2_]^+^	1621.6944	1621.7011

## Conclusions

A resin displaying a disulfide-containing apo-DFOB probe enriched NiSOD from the *S. pilosus* proteome, which was identified to 20% coverage from a pulldown-proteomics workflow. NiSOD was enriched on the apo-DFOB-displaying resin, and not on the Fe(iii)-DFOB- or methylamine-displaying resins. The system showed high non-specific binding, likely due to the indiscriminatory displacement of the probe ligands from the resin by proteins containing surface exposed Cys residues. Although the dithiol-cleavable resin system has appeal in the potential to enable the reduction-mediated release of probe-protein complexes, the Cys-based non-specific binding prescribes the need for bulk proteome alkylation or a modified resin design.^54^ NiSOD from *Streptomyces* sp. does not contain any Cys residues besides the two involved in Ni(ii) coordination, which may have been a factor that contributed to the enrichment observed in this study.

The results suggested that the apo-DFOB-displaying resin might retain NiSOD *via* the formation of a ternary hexadentate complex, with one hydroxamic acid unit contributing to the tetradentate S2N2-Ni(ii) coordination sphere. This binding mode would not be possible for coordinatively-saturated Fe(iii)-DFOB or the non-coordinating methylamine control system. Support for the proposed DFOB-NiSOD ternary complex was provided from experimental ESI-TOF-MS measurements on a model system. Although any functional significance of a DFOB-NiSOD complex is unclear, the study shows siderophores could be involved in modulating metal-dependent processes in biology beyond their established roles in iron uptake.

## Experimental

### Materials and methods

Deferoxamine mesylate (≥92.5%), methylamine hydrochloride (≥99%), 3,3′-dithiodipropionic acid di(*N*-hydroxysuccinimide ester), triethylamine (≥99.5%), tris(2-carboxyethyl)phosphine hydrochloride, sodium hydroxide (≥98%), sodium chloride (≥99%), potassium chloride (≥99%), sodium phosphate dibasic (≥98%), and iron(iii) chloride hexahydrate (97%), Chelex® resin, Trizma base (≥99.9%), l-threonine (≥98%), zinc sulfate heptahydrate (≥99.8%), iron(iii) perchlorate hydrate (≥99.9%), urea (≥99%), thiourea (≥99%), iodoacetamide (≥99%), Roche EDTA-free protease inhibitor cocktail, triethylammonium bicarbonate, trifluoroacetic acid (≥99%), porcine pancreas trypsin (sequencing grade) and *N*,*N*-dimethylformamide (≥99.8%) were purchased from Sigma Aldrich. 4-(2-Hydroxyethyl)-1-piperazineethanesulfonic acid (HEPES; 99%) was purchased from Astral Scientific. Dichloromethane, diethyl ether, methanol, magnesium sulfate heptahydrate (≥98%), calcium chloride (≥97%), potassium phosphate monobasic (≥99%), and acetone (99%) were obtained from Ajax Finechem. Acetonitrile (≥99.9%) was purchased from Merck, and N_2_ gas was obtained from BOC. Ni(ii)-IDA IMAC columns were purchased from GE Healthcare. Ultrapure water was prepared using a Millipore Q-pod system. CarboxyLink^TM^ Coupling Resin and sulfosuccinimidyl 6-(3′-(2-pyridyldithio)propionamido)hexanoate (Sulfo-LC-SPDP; ≥90%) were purchased from ThermoFisher. *Streptomyces pilosus* (ATCC 19797) was supplied from the American Type Culture Collection. YM broth was purchased from Difco. Acetic acid was purchased from BioLab, and Oasis hydrophilic lipophilic balanced cartridges were obtained from Waters.

### Instrumentation

#### LC-MS (compound purification)

Reverse-phase LC-MS was performed using an Agilent Technologies HPLC system consisting of an autosampler (100 μL loop), an Agilent 1260 Infinity Degasser, a quaternary pump, a fraction collector, and an Agilent 6120 Series Quadrupole electrospray ionisation mass spectrometer. A reverse-phase Agilent pre-packed C18 column (2.1 × 150 mm, i.d., 0.3 mL min^−1^, particle size 3.5 μm) was used for all experiments. The following instrument conditions were used: 5 μL injection volume with 3 kV spray voltage, 3.5 kV capillary voltage, 350 °C capillary temperature, and a 10 V tube lens-offset. The mobile phase consisted of acetonitrile and formic acid (99.9 : 0.1) (mobile phase B), and H_2_O and formic acid (99.9 : 0.1) (mobile phase A). The method used a 5–95% B:A gradient over 20 min, with a flow rate of 0.2 mL min^−1^. Samples prepared for LC-MS were dissolved in water to approximately 1–10 mg mL^−1^. Insoluble material was removed with a syringe filter (PTFE filter, 0.45 μm pore size) purchased from AllPure. Spectral data was acquired and processed using Agilent OpenLAB Chromatography Data System ChemStation Edition.

#### Flash chromatography

Flash chromatography was performed on a Grace Reveleris X2 autoflash system using a Buchi FlashPure C18 cartridge (4 g, particle size 30 μm). The method used a mobile phase of 20–80% acetonitrile:water gradient over 45 min, with a flow rate of 10 mL min^−1^. The elution of compounds of interest were UV-monitored at 220 and 280 nm.

#### LC-MS (proteomics)

Reverse-phase LC-MS was performed using a Dionex UltiMate 3000 UHPLC system and a Dr Maisch ReproSil-Pur 120 C18-AQ column (30 cm × 75 μm (i.d.), 300 nL min^−1^, particle size 1.9 μm). The mobile phase consisted of a gradient of 5–35% acetonitrile with 0.1% formic acid over 45 min. The UHPLC system was coupled to a ThermoFisher Q-Exactive HFX mass spectrometer. Data was acquired in positive polarity, data-dependent acquisition mode, with MS1 scans acquired from 300–5000 *m*/*z* (60 000 resolution, 50 msec injection time), followed by 10 MS/MS scans performed using higher-energy collisional dissociation fragmentation (0.7 *m*/*z* isolation window, 15 000 resolution, 30 normalised collision energy).

### Molecular modelling

A 3-mercaptopropanoic acid unit was appended to the N-terminus of DFOB (built in a linear form) to give 2 and the hydroxamic acid group proximal to the *N*-acetyl group was bound to the coordination site of NiSOD (PDB: 1t6u).^24^ The structure was minimised in HyperChem (Version 8.0) with no constraints. The RMS between native NiSOD and the NiSOD region of the model was 3.393 Å. Images were generated using the Swiss PBD Viewer.

### Synthesis and characterisation

#### *N*-DFOB-3-mercaptopropanamide (DFOB-SH) (2)

Triethylamine (26.0 μL, 0.186 mmol) was added to DFOB mesylate (107.2 mg, 0.163 mmol) and DTSP (30 mg, 0.074 mmol) in DMF (6 mL), and the reaction solution was stirred at 70 °C for 20 h to yield the intermediate dithiobis(*N*-DFOB-propanamide) ((DFOB)_2_-SS) (2a), which was confirmed from LC-MS measurements, but not isolated. The solvent was removed *in vacuo*, and the solid was resuspended in water and the solution was adjusted to pH 10. TCEP (0.111 mmol) was added to the mixture and the vessel was nitrogen-purged, then stirred at room temperature for 4 h. Upon completion, the solvent was removed *in vacuo* to leave a white solid (98.9 mg, 56.5%). An aliquot of the crude DFOB-SH reaction mixture (5 mg, pH 8.9) was syringe-filtered (PTFE filter, 0.45 μm pore size) and the flowthrough adsorbed onto Ni(ii)-IDA IMAC columns (column volume (CV) = 1 mL). The resin matrix was washed with 5 CV binding buffer (0.025 mM HEPES, 0.5 M NaCl, pH 8.9) to remove non-Ni(ii) coordinating species. Ni(ii)-coordinating compounds were subsequently eluted with 5 CV elution buffer (0.025 mM HEPES, 0.5 M NaCl, pH 5.5).

#### *N*-Methyl-3-mercaptopropanamide (MA-SH) (3)

Methylamine hydrochloride (7.35 mg, 0.109 mmol) and DTSP (20 mg, 0.050 mmol) were dissolved in a miscible solution of water and acetone (5.5% v/v water in acetone). Triethylamine (17.3 μL, 0.124 mmol) was added and the reaction solution was stirred at room temperature for 20 h to yield dithiobis(*N*-methyl-propanamide) ((MA)_2_-SS) (3a). TCEP (0.074 mmol) was added to the mixture and the reaction was nitrogen-purged, then stirred at room temperature for 4 h. Upon completion, the solvent was removed *in vacuo*. Purification was performed by flash chromatography.

#### Preparation of DFOB-SS-Resin (2-R), Fe(iii)-DFOB-SS-Resin (Fe(iii)-2-R) and MA-SS-Resin (3-R)

An aliquot of CarboxyLink^TM^ Coupling Resin (resin bed volume (RBV) = 150 μL) was incubated with 12.7 μmol Sulfo-LC-SPDP for 1.5 h with gentle inversion to generate the pyridyldithiol-activated resin. The column was drained and equilibrated with 10 RBV of PBS/EDTA (0.137 M NaCl, 2.7 mM KCl, 10 mM Na_2_HPO_4_, 1.8 mM KH_2_PO_4_, 1 mM EDTA, pH 10.0). An aliquot (25.4 μmol) of syringe-filtered (PTFE filter, 0.45 μm pore size) DFOB-SH (2) or MA-SH (3) was added to the column and mixed by gentle inversion for 45 min to form DFOB-SS-Resin (2-R) or MA-SS-Resin (3-R) with a coupling efficiency, determined from the concentration of pyridine-2-thione in the flow-through, of 32% or 42%, respectively. Fe(iii)-DFOB-SS-Resin (Fe(iii)-2-R) was prepared by the addition of 50 μmol FeCl_3_ to the 2-R system. Resins were stored in PBS/EDTA at 4 °C until required. To elute disulfide-immobilised compounds, the resin was incubated with 12.7 μmol of TCEP for 20 min with gentle inversion.

### Proteomics methods

#### Culturing and proteome production

*S. pilosus* precultures were prepared in YM broth (2.1% w/v). Adventitious iron was removed by stirring with Chelex® resin (1g per 100 mL) for 3 h, which was subsequently removed by decantation. The media was inoculated with *S. pilosus* under sterile conditions and incubated for 4 d (28 °C, 160 rpm). Cells were harvested by centrifugation (3500 rpm, 7 min), and resuspended in an enriched medium consisting of YM broth (4.2% w/v), phosphate buffer and a solution of enrichment components in a 2 : 1 : 1 volumetric ratio. The YM broth and phosphate buffer (0.94 M KH_2_PO_4_, 140 mM Na_2_HPO_4_) were prepared with Chelex®. The solution containing enrichment components (400 mM Trizma base, 54.4 mM CaCl_2_, 9.72 mM MgSO_4_, 3.36 mM threonine, 55.6 μM ZnSO_4_) was sterile filtered (Minisart, polyethersulfone filter, 0.2 μm pore size) prior to its addition. *S. pilosus* cultures were incubated at 28 °C and 160 rpm for 10 days. Samples of culture supernatant (1 mL) were taken at days 0, 2, 4, 6, 8, and 10. Siderophore production was monitored spectrophotometrically by Fe(iii)-addition assays.

#### Fe(iii) addition assay

Ferric assay solution (100 μL, 10 mM ferric perchlorate in 0.2 M HCl) was added to culture supernatant (200 μL), and the absorbance at 470 nm was measured after 5 min using a BMG Labtech FLUOstar Omega spectrophotometer.

#### Preparation of protein lysate

Cells were harvested by centrifugation (3220 rpm, 25 min, 4 °C), washed twice in Tris buffer (10 mM Tris, 10 mM NaCl, pH 7.5), and frozen at −20 °C until required. To liberate proteins, cells were resuspended in 10 mL of Tris buffer containing EDTA-free cOmplete™ protease inhibitors and disrupted in 500 μL aliquots under ice cooling (6 × 10 s on, 6 × 30 s off). A Crown Scientific Ultrasonic Processor was used with 20% of the maximum power. Cell debris was removed by centrifugation (15 000 rpm, 15 min, 4 °C) and the supernatant was collected.

#### Pulldown procedure

30 μL of resin (DFOB-SS-Resin (2-R), Fe(iii)-DFOB-SS-Resin (Fe(iii)-2-R), or MA-SS-Resin (3-R)) were each incubated with 650 μL of *S. pilosus* protein lysate (∼1.6 mg mL^−1^) for 2.5 h at 4 °C with gentle inversion. The flow-through was collected, and the resin was washed five times with 650 uL Tris buffer until no protein was detected by a Bradford assay. Proteins bound to the probe were eluted by incubation with 25.4 μmol TCEP for 20 min. The eluent was collected, and the resin was washed twice with 150 μL Tris buffer. Protein concentration was estimated by a Bradford assay.^55^

#### Proteomics sample preparation

Proteins from affinity pull-down experiments were extracted by chloroform-methanol precipitation,^56^ then reduced and alkylated with 10 mM DTT and 20 mM iodoacetamide. Protein samples (10 μg) were run at 200 V on an SDS-PAGE gel (4–12% polyacrylamide), fixed in a solution of methanol:acetic acid (10 : 7% v/v in MilliQ) and stained with Coomassie Blue. Protein bands selected from the 1-DE gel were excised for proteolytic digestion and suspended in 50 mM triethylammonium bicarbonate (pH 7.6). Sequencing grade porcine pancreas trypsin (12 ng) was incubated with each sample overnight at 37 °C. Digests were acidified to 1% trifluoroacetic acid before desalting and concentration by hydrophilic-lipophilic balance chromatography. Peptides were analysed by LC-MS. Densitometry measurements were conducted using FIJI Image-J.

#### Proteomics data analysis

Data was processed against the bacterial SwissProt database (October 2022) using a Mascot server. Searches were performed permitting up to 2 missed cleavages; mass tolerance 10 ppm (MS1) and 0.1 Da (MS2); and variable modifications (oxidation (Met), acetylation (protein N-termini), and carbamidomethyl (Cys)). Proteins present in numerous (>3) unrelated protein bands were eliminated as candidates, and the remaining proteins was selected based on score, peptide matches and sequence coverage.

### NiSOD expression and purification

The plasmid pET-22b-pelB-NiSOD was transformed into *Escherichia coli* DL41 (DE3) and expressed in 0.5 L LB containing 0.1% glucose and 1 mM MgSO_4_ as described except that protein expression was induced with 0.5 mM IPTG at OD_600_ = 1.8.^57^ After induction, cells were harvested by centrifugation (4000 rpm for 5 min). The resulting cell pellet was resuspended in 50 mL of osmotic shock buffer (30 mM Tris·HCl, pH 8.0, 20% sucrose, 1 mM EDTA) with shaking at 20 °C for 15 min. The re-suspended cells were then centrifuged at 4000g for 10 min. The supernatant was retained and the pellet re-suspended in 50 mL ice-cold 5 mM MgSO_4_. The resuspended cells were kept on ice for 10 min with brief agitation every 2 min, then centrifuged at 4500 rpm for 20 min. The supernatant was retained and the pellet discarded. The two supernatants were then combined, brought to 90% ammonium sulfate saturation, stirred at room temperature for 2 h and centrifuged. The pellet was resuspended in 20 mM Tris, pH 8.0, 2 M ammonium sulfate, to which TCEP was added to 5 mM final concentration, followed by 1 mM NiCl_2_ to final concentration upon which the sample turned an orange/brown colour indicative of Cys reduction and generation of nickelated SOD. The sample was then purified over hydrophobic interaction (phenyl sepharose; Amersham Biosciences) eluting with 20 mM Tris, pH 8.0. The eluate was analysed by SDS-PAGE to confirm purity and the protein concentration was calculated using *ε*_278_ = 21.3 × 10^3^ M^−1^ cm^−1^.^58^

### UV-visible spectroscopy from solutions of 1 and NiSOD

NiSOD was desalted into assay buffer (20 mM HEPES, pH 7.0, 200 mM KCl) using a PD-10 column and protein concentration determined. A freshly prepared solution of 60 mM DFOB was prepared in the assay buffer. NiSOD was diluted 1 : 10 (15 μM final concentration) and 200 μL aliquots were pipetted into wells of a UV-transparent microtitre plate (Corning). 90 μL of DFOB was mixed with 10 μL of protein (1 : 10 dilution) and this was mixed with the first 200 μL well. Then 100 μL of the mixture was extracted and pipetted into the next well to generate a series of 8 DFOB concentrations from 0.024 mM to 18 mM and a 0 mM DFOB control. A control dilution series of DFOB plus 200 μL buffer was generated in the same way except that 90 μL DFOB was mixed with 10 μL of buffer only. Samples were incubated at 22 °C for 10 min then scanned from 280 to 600 nm at 1 nm intervals (Thermofisher Multiskan GO) in precision mode.

### ESI-TOF-MS measurements

The NiSOD model peptide (HisCysAspLeuProCysGlyValTyr-NH_2_) was purchased from Synpeptide Co. (Shanghai, China) and its exact concentration was determined by pH-potentiometric titration. The NiCl_2_ stock solution was prepared from the highest available grade (≥99.95%; VWR Int., USA) and its concentration was determined by complexometric titration using EDTA. All experiments used doubly-deionized and/or ultrafiltered water (ELGA Purelab Classic system). ESI-TOF-MS measurements were made with a Bruker maXis II MicroTOF-Q type Qq-TOF-MS instrument (Bruker Daltonik, Bremen, Germany) in positive ion mode. The instrument was equipped with an electrospray ion source where the spray voltage was 4 kV. N_2_ was used as a drying gas and the drying temperature was 200 °C. The spectra were accumulated and recorded using a digitalizer at a sampling rate of 2 GHz. The mass spectra were calibrated externally using the exact masses of sodium formate clusters. The spectra were evaluated using DataAnalysis 4.4 software from Bruker. The isotopic patterns of the complexes were calculated using the IsotopePattern software from Bruker or ChemCalc.^59^

## Data availability

All relevant data are presented in the main text and ESI.†

## Author contributions

The study was conceptualized by RC. All authors contributed to the design of the study methods and the analysis of results. Experimental data was generated by JN, JLW, MYW, NL, TEM, JW, and PTC. The first draft of the manuscript was written by RC and JN, with all authors contributing to manuscript review, editing, and data presentation, and approving the final submission.

## Conflicts of interest

There are no conflicts of interest to declare.

## Supplementary Material

CB-004-D3CB00097D-s001
