# Supplementary material for: Reduction-cleavable desferrioxamine B pulldown system enriches Ni(ii)-superoxide dismutase from a Streptomyces proteome
Source: RSC Chem Biol. 2023 Oct 2;4(12):1064–72. doi: 10.1039/d3cb00097d (PMC10685849; doi:10.1039/d3cb00097d)
Supplement: CB-004-D3CB00097D-s001 [file CB-004-D3CB00097D-s001.pdf]

Electronic Supplementary Material (ESI) for RSC Chemical Biology.
This journal is © The Royal Society of Chemistry 2023

Supporting Information

Reduction-cleavable desferrioxamine B pulldown system enriches Ni(II)-superoxide dismutase from a *Streptomyces* proteome

Table of contents

Item **Pg**

Table of contents……………………………………………………………………………………………... S1

Scaled synthesis and characterisation protocols………………………………………………………... S2

Figure S1. ^{1}H NMR (600 MHz, DMSO-d$_6$) spectrum of **2** ………………………………………… S3

Figure S2. ^{13}C{^{1}H} NMR (150 MHz, DMSO-d$_6$) spectrum of **2**……………………………………… S3

Figure S3. ^{1}H-^{1}H COSY NMR (600 MHz, DMSO-d$_6$) spectrum of **2**………………………………… S4

Figure S4. ^{1}H-^{13}C NMR HSQC (600 MHz, DMSO-d$_6$) spectrum of **2**……………………………… S4

Figure S5. ^{1}H-^{13}C NMR HMBC (600 MHz, DMSO-d$_6$) spectrum of **2** ……………………………… S5

Figure S6. HRMS (ESI)$^+$ spectrum of **2**…………………………………………………………………… S5

Figure S7. ^{1}H NMR (600 MHz, DMSO-d$_6$) spectrum of **3a** and **3** ………………………………… S6

Figure S8. ^{13}C{^{1}H} NMR (150 MHz, DMSO-d$_6$) spectrum of **3a** and **3**…………………………… S6

Figure S9. ^{1}H-^{1}H COSY NMR (600 MHz, DMSO-d$_6$) spectrum of **3a** and **3**……………………… S7

Figure S10. ^{1}H-^{13}C NMR HSQC (600 MHz, DMSO-d$_6$) spectrum of **3a** and **3**……………………… S7

Figure S11. ^{1}H-^{13}C NMR HMBC (600 MHz, DMSO-d$_6$) spectrum of **3a** and **3**……………………… S8

Figure S12. LC traces and experimental and calculated MS isotope patterns from **2a** and **2**……………… S9

Figure S13. LC traces and experimental and calculated MS isotope patterns from **3a** and **3**……………… S9

Figure S14. Electronic spectra from solutions of Fe(III)-**1** and Fe(III)-**2**………………………………… S9

Figure S15. Experimental and calculated MS isotope patterns from a solution of Fe(III)-**2**………………… S9

Figure S16. Fe(III) additional assay on sub-samples of *S. pilosus* culture ……………………………… S10

Figure S17. Protein content (Bradford method) in fractions from pulldown procedure using **2-R**, Fe(III)-**2-R** or **3-R**…………….. S10

Figure S18. SDS-PAGE gel from pulldown procedure……………………………………………………… S10

Figure S19. SDS-PAGE gel from pulldown procedure with marked regions analysed using densitometry ……………………….. S10

Figure S20. MS/MS spectrum of peptides from NiSOD identified from the pulldown procedure…….……… S11

Figure S21. UV-Vis spectra from titration of **1** with recombinant NiSOD……………………………………… S11

Table S1. MS-MS peptide reads for NiSOD………………………………………………………………… S12

Scaled synthesis and characterisation protocols

NMR spectroscopy (compound characterisation). ^{1}H and ^{13}C spectra were acquired on a 600 MHz Bruker Avance III NMR spectrometer equipped with a TCI cryoprobe at 600 MHz and 150 MHz respectively. Spectra were recorded at 298 K using DMSO-d_6 as the solvent and internal lock. All spectra were referenced to the residual solvent signal (DMSO-d_6: ^{1}H 2.50 ppm, ^{13}C 39.52 ppm) and are reported as follows: (1) chemical shift (ppm), (2) integration, (3), multiplicity (s, singlet; brs, broad singlet; d, doublet; dd, doublet of doublets t, triplet; m, multiplet); (4) coupling constant (Hz). Signals were assigned to individual compounds with the aid of 2D homonuclear (^{1}H-^{1}H COSY) and heteronuclear (^{1}H-^{13}C HSQC and HMBC) correlation spectroscopy.

HRMS (compound characterisation). HRMS analysis was conducted on a Thermofisher Vanquish Horizon UHPLC coupled to a Thermofisher Q-Exactive HFX Hybrid Quadrupole-Orbitrap mass spectrometer. Solvent A was 0.1% formic acid in water and solvent B was 0.1% formic acid in acetonitrile. An Agilent Zorbax Eclipse XDB-C18 column (150 mm x 2.1 mm I.D., 3.5 µm) maintained at 30 °C with a constant flow rate of 0.2 mL/min was employed. A sample injection volume of 5 µL was used and the solvent program was as follows: a linear gradient of 5-95% solvent B over 25 min followed by 95% solvent B held for 5 min. Solvent B was then held at 5% for an additional 5 min to re-equilibrate for the following injection. The mass spectrometer was operated in positive ion-mode using electrospray ionisation (ESI) with a mass range of 100-1500 m/z. A spray voltage of 3.5 kV was used and the capillary temperature maintained at 300 °C.

HPLC (compound purification). Preparative HPLC purification was undertaken on a Shimadzu LC-20 series high performance liquid chromatography system with two LC-20AP preparative pumps, a SIL-10AP autosampler and a SPD-20A photo-diode array detector. A Shim-pack GIS-C18 column (150 x 20 ID, 5 µm) was used at 20 mL/min. Mobile phase A was 0.1 % formic acid in Milli-Q water and mobile phase B was 0.1 % formic acid in acetonitrile with solvent programs as stated below for individual compounds.

Preparative scale synthesis of *N*-DFOB-3-mercaptopropanamide (DFOB-SH) (2). Triethylamine (67 µL, 0.48 mmol.) and DTSP (53 mg, 0.13 mmol.) were added to a solution of DFOB mesylate salt (151 mg, 23 mmol.) in anhydrous DMF (2 mL) under nitrogen. The resulting mixture was heated at 70 °C for 16 h under nitrogen. The solvent was removed in vacuo and the crude material reconstituted in water. The suspension was pH adjusted to 10 with 1 M sodium hydroxide solution. TCEP hydrochloride (0.5 mL, 0.5 M aqueous solution, 0.25 mmol.) was added to the aqueous mixture and left to stir at room temperature under nitrogen for 4 h. The solvent was removed in vacuo and the crude material reconstituted in 1 M sodium hydroxide solution for purification by preparative HPLC (5-60 % mobile phase B over 20 min) yielding the desired product **(2)** as a white powder (50 mg, 34%).

N-DFOB-3-mercaptopropanamide (DFOB-SH) **(2)**. ^{1}H NMR (600 MHz, DMSO-d_6) δ 9.67 (1H, brs), 9.63 (2H, brs), 7.84 (1H, dd, J = 4.8, 5.4 Hz), 7.77 (2H, dd, J = 4.8, 5.4 Hz), 3.45 (6H, t, J = 6.7 Hz), 2.98-3.03 (6H, m), 2.64 (2H, t, J = 6.6 Hz), 2.57 (4H, t, J = 7.1 Hz), 2.35 (2H, t, J = 6.6 Hz), 2.26 (4H, t, J = 7.3 Hz), 1.96 (3H, s), 1.49 (6H, m), 1.38 (6H, m), 1.22 (6H, m). ^{13}C (150 MHz, DMSO-d_6) δ 171.97, 171.32, 170.14, 170.01, 47.08, 46.79, 38.43, 38.38, 29.91, 28.82, 27.58, 26.04, 23.50, 20.36, 20.01. HRMS (ESI)$^+$ calculated for $C_{28}H_{53}N_6O_9S$ $[M+H]^+$: 649.3589, observed: 649.3580.

Semi-preparative scale synthesis of *N*-methyl-3-mercaptopropanamide (MA-SH) (3). Methylamine hydrochloride (35 mg, 0.52 mmol) and DTSP (105 mg, 0.26 mmol) was dissolved in a miscible solution of 5.5% water in acetone. Triethylamine (72 µL, 0.25 mmol) was added and the reaction stirred at room temperature for 24 h. The solution was then concentrated in vacuo and redissolved in water. 2-Mercaptoethanol (36 µL, 0.52 mmol) was then added into the crude mixture and the reaction stirred under nitrogen overnight. The resulting solution was then HPLC purified (0% mobile phase B held for 3 min, 0-20% mobile phase B over 12 min) and the collected fractions dried in vacuo. Qualitative LC-MS analysis confirmed the isolation of the desired free thiol however, NMR analysis showed MA-SH **(3)** dimerised on standing to regenerate $(MA)_2$-SS **(3a)**. The isolated material was spectroscopically characterised as a mixture of $(MA)_2$-SS **(3a)** and MA-SH **(3)**.

3,3'-Disulfanediylbis(N-methylpropanamide) $(MA)_2$-SS **(3a)**. ^{1}H NMR (600 MHz, DMSO-d_6) δ 7.87 (2H, brs), 2.87 (4H, t, J = 7.2 Hz), 2.57 (3H, d, J = 4.6 Hz), 2.44 (4H, t, J = 7.2 Hz). ^{13}C (150 MHz, DMSO-d_6) δ 170.44, 34.91, 33.87, 25.46.

N-Methyl-3-mercaptopropanamide (MA-SH) **(3)**. ^{1}H NMR (600 MHz, DMSO-d_6) δ 7.86 (1H, brs), 2.63 (2H, dd, J = 7.2, 14.4 Hz), 2.57 (3H, d, J = 4.6 Hz), 2.35 (2H, t, J = 7.2 Hz), 2.27 (1H, t, J = 8.0 Hz). ^{13}C (150 MHz, DMSO-d_6) δ 170.65, 38.28, 25.53, 19.97.

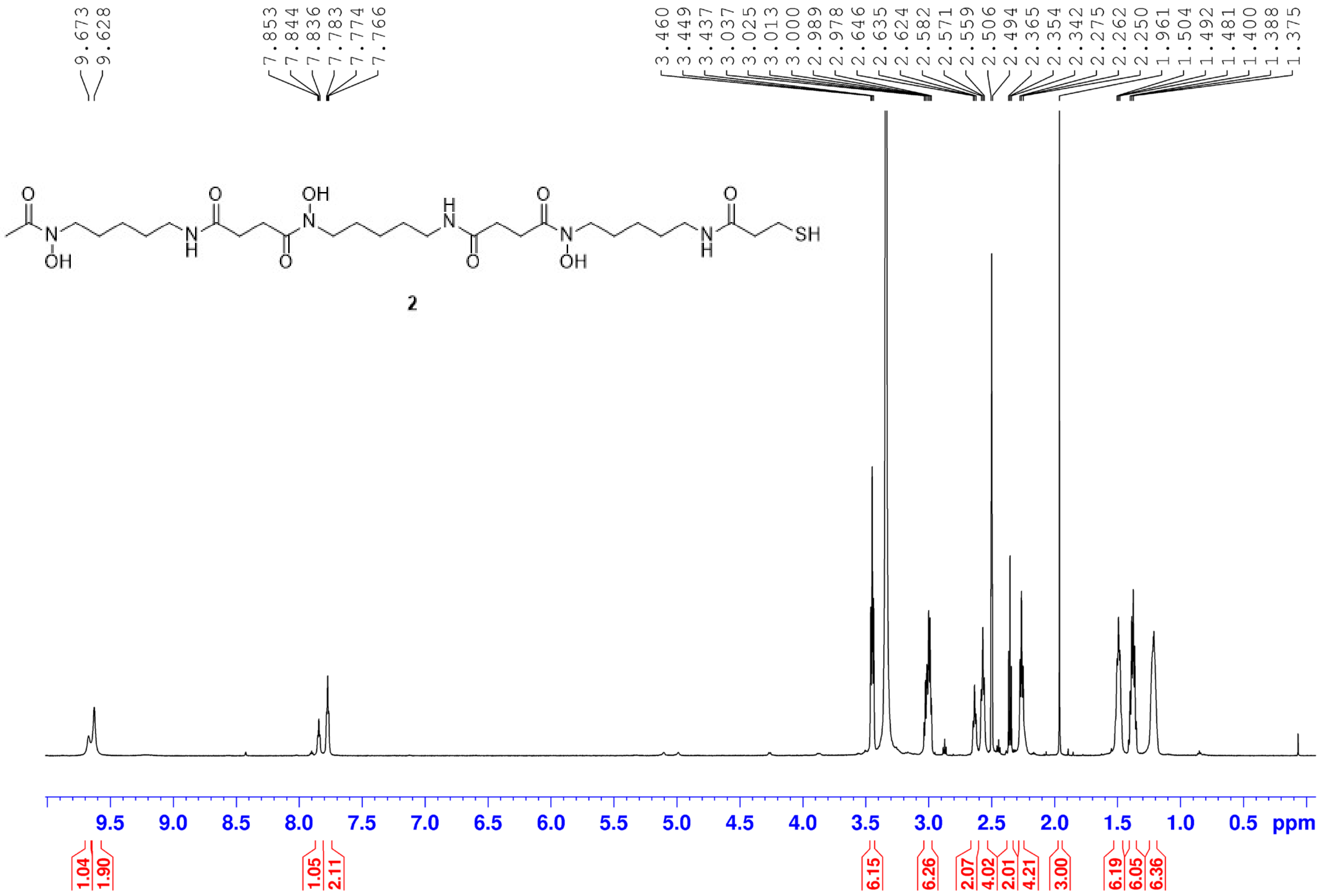

Fig. S1. ^{1}H NMR (600 MHz, DMSO-d$_6$) spectrum of **2**

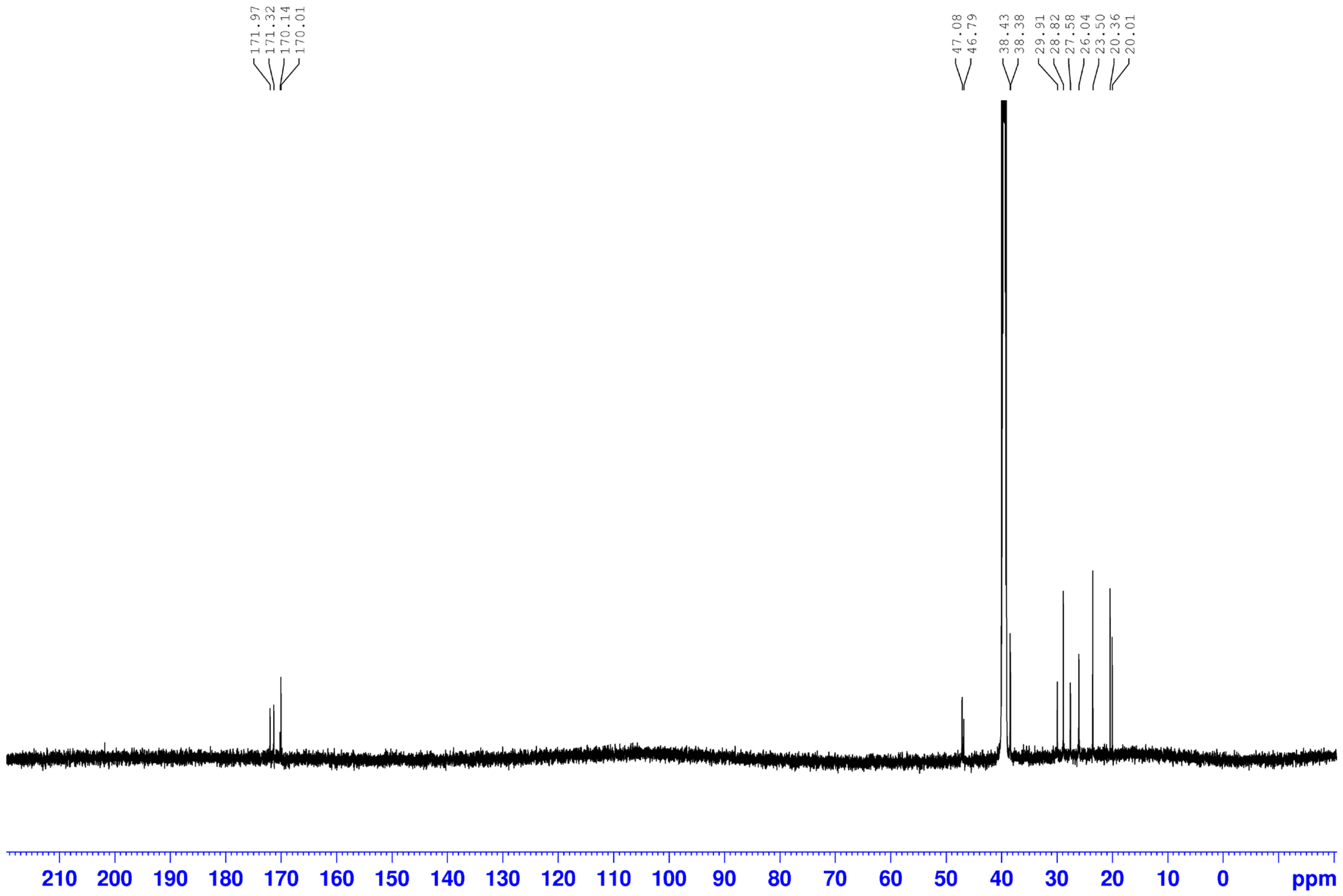

Fig. S2. ^{13}C{^{1}H} NMR (150 MHz, DMSO-d$_6$) spectrum of **2**

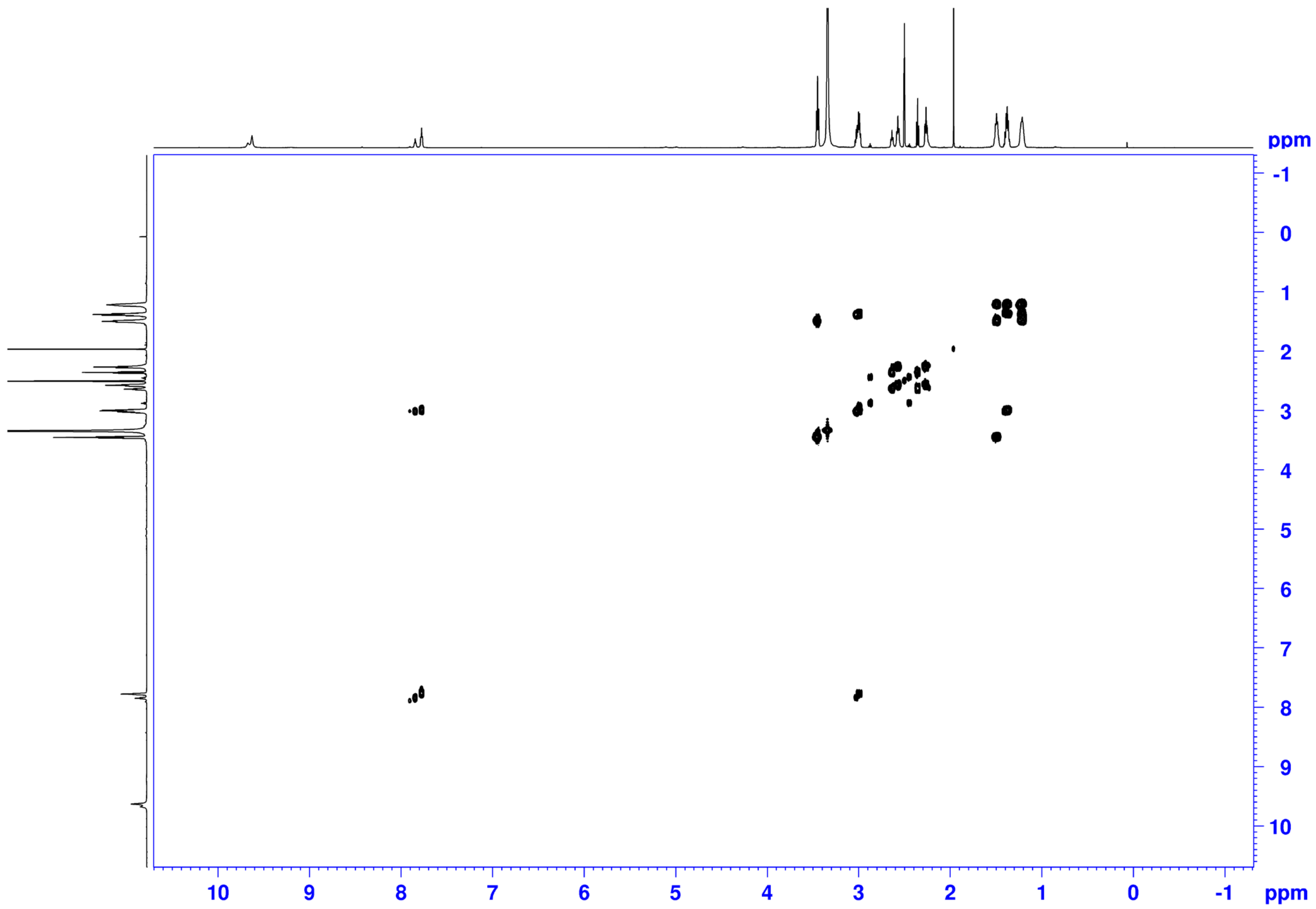

Fig. S3. ^{1}H-^{1}H COSY NMR (600 MHz, DMSO-d_6) spectrum of **2**

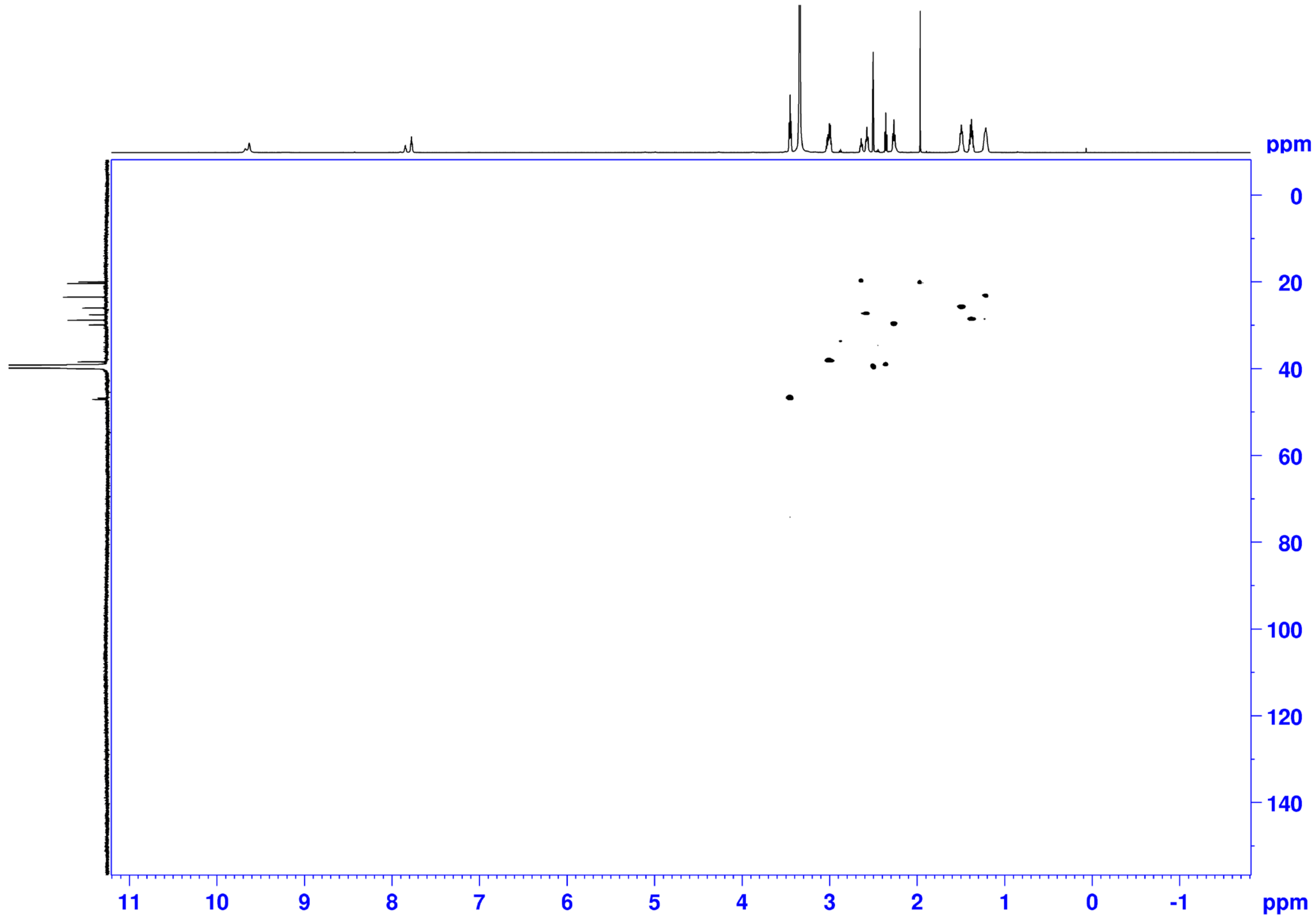

Fig. S4. ^{1}H-^{13}C HSQC NMR (600 MHz, DMSO-d_6) spectrum of **2**

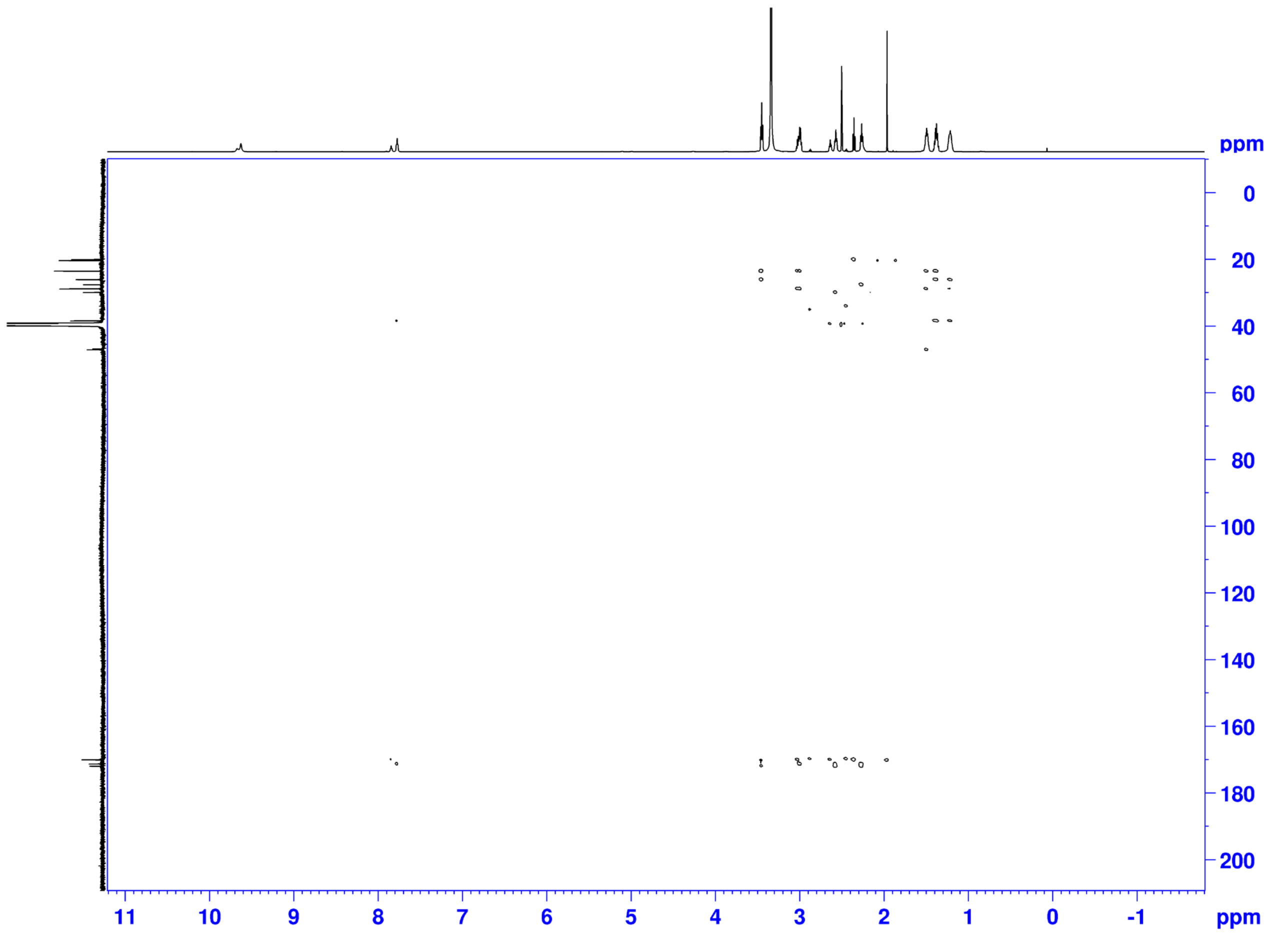

Fig. S5. ^{1}H-^{13}C HMBC NMR (600 MHz, DMSO-d_6) spectrum of **2**

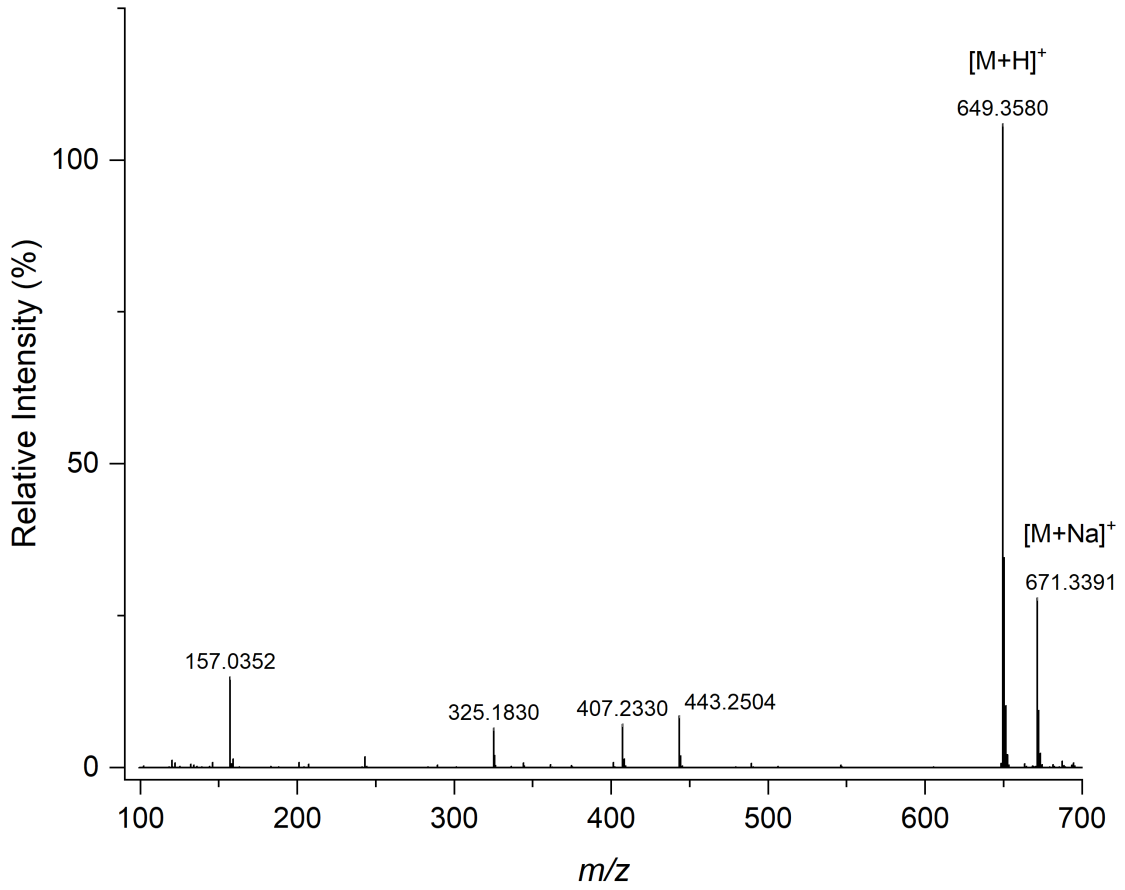

Fig. S6. HRMS (ESI)$^{+}$ spectrum of **2**

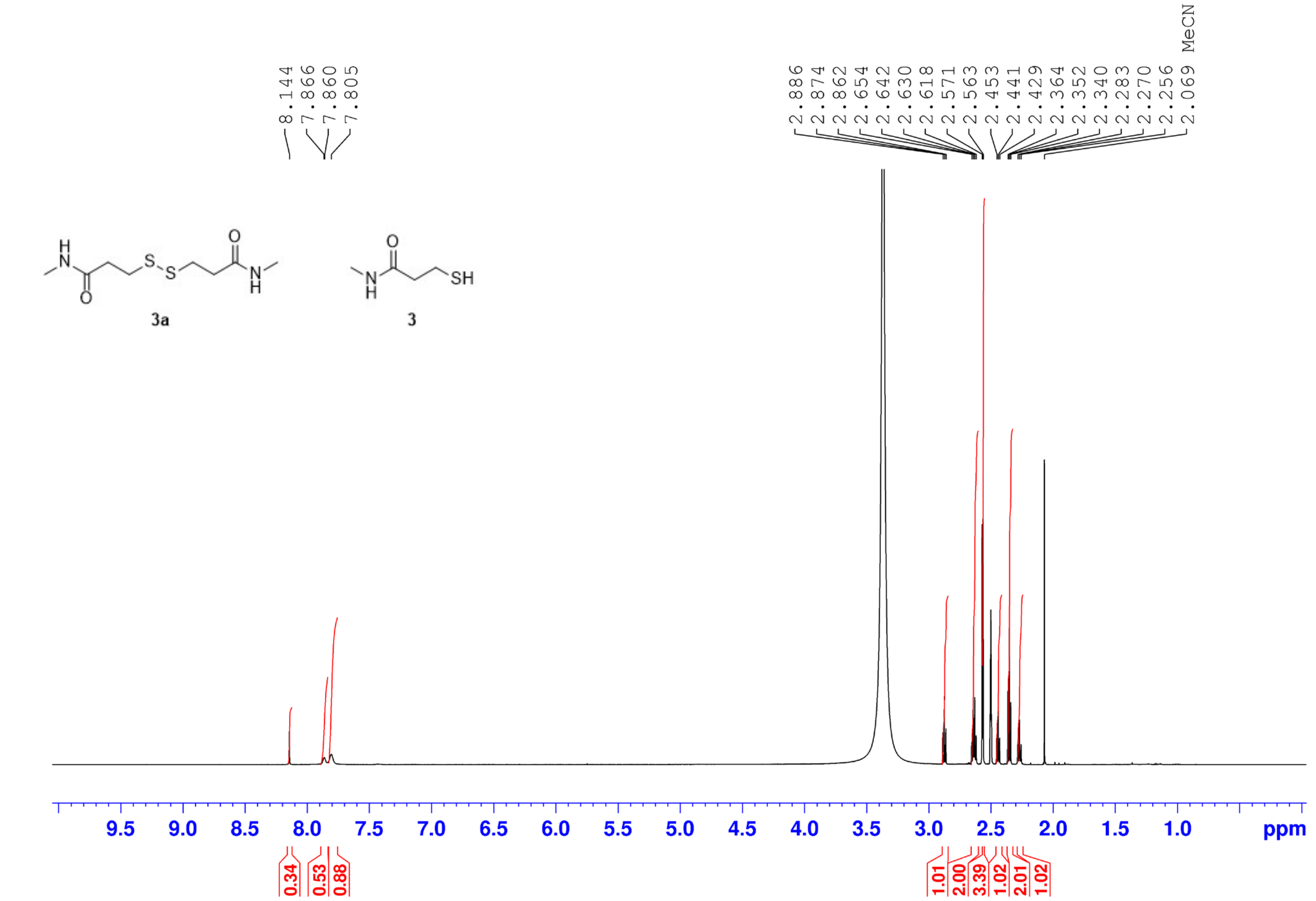

Fig. S7. ^{1}H NMR (600 MHz, DMSO-d_6) spectrum of **3a** and **3**

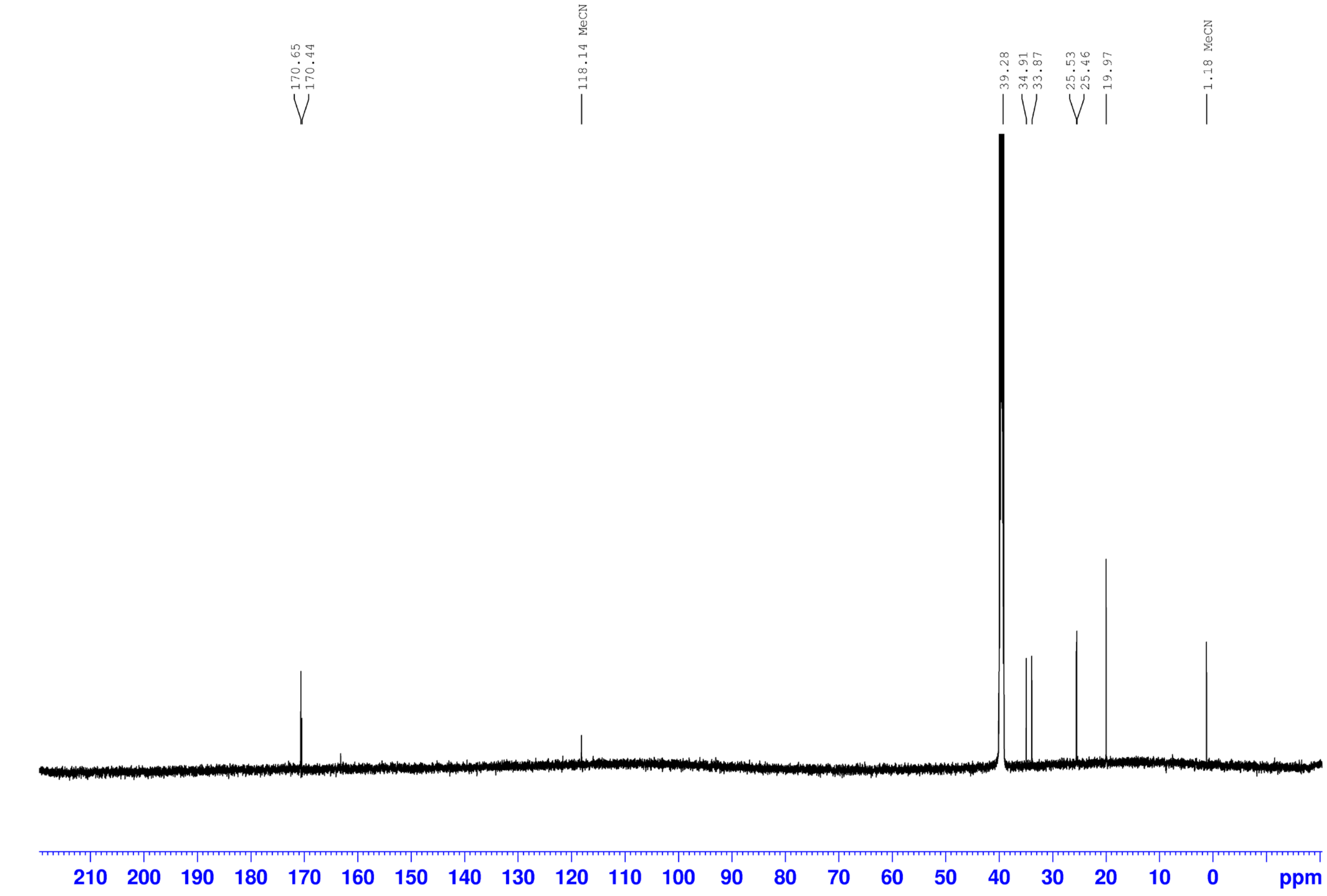

Fig. S8. $^{13}C\{^{1}H\}$ NMR (150 MHz, DMSO-d_6) spectrum of **3a** and **3**

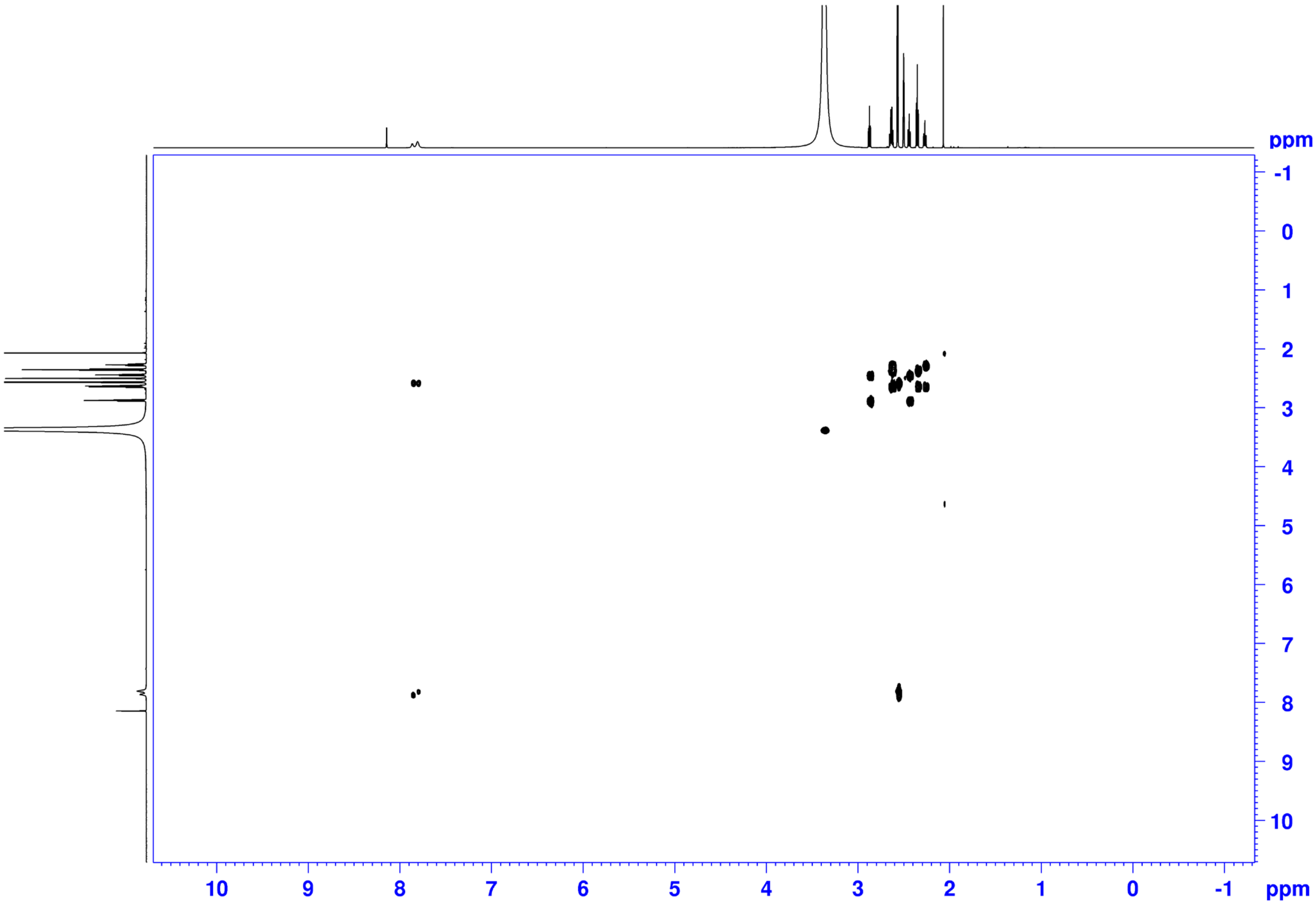

Fig. S9. ^{1}H-^{1}H COSY NMR (600 MHz, DMSO-d$_6$) spectrum of **3a** and **3**

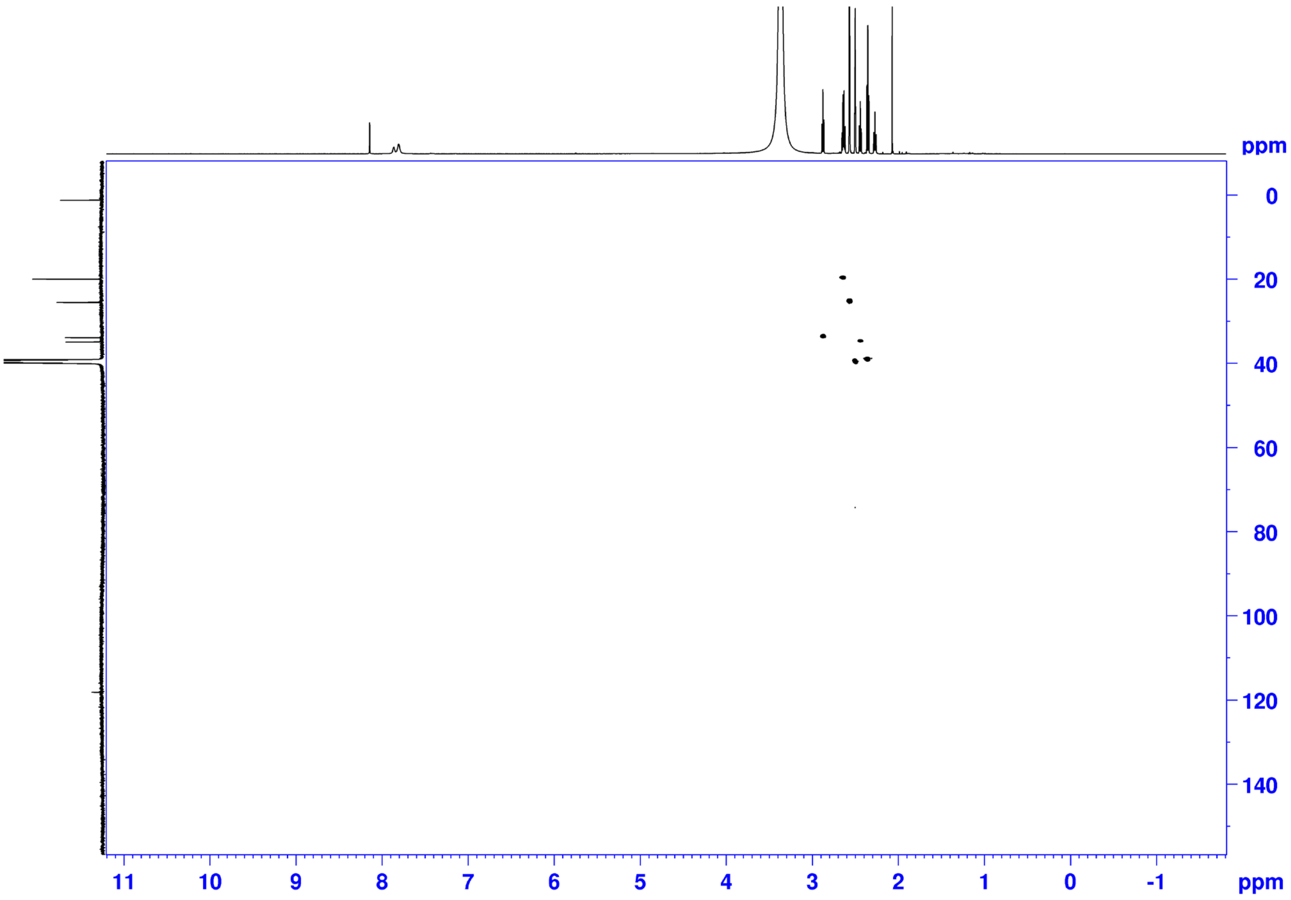

Fig. S10. ^{1}H-^{13}C HSQC NMR (600 MHz, DMSO-d$_6$) spectrum of **3a** and **3**

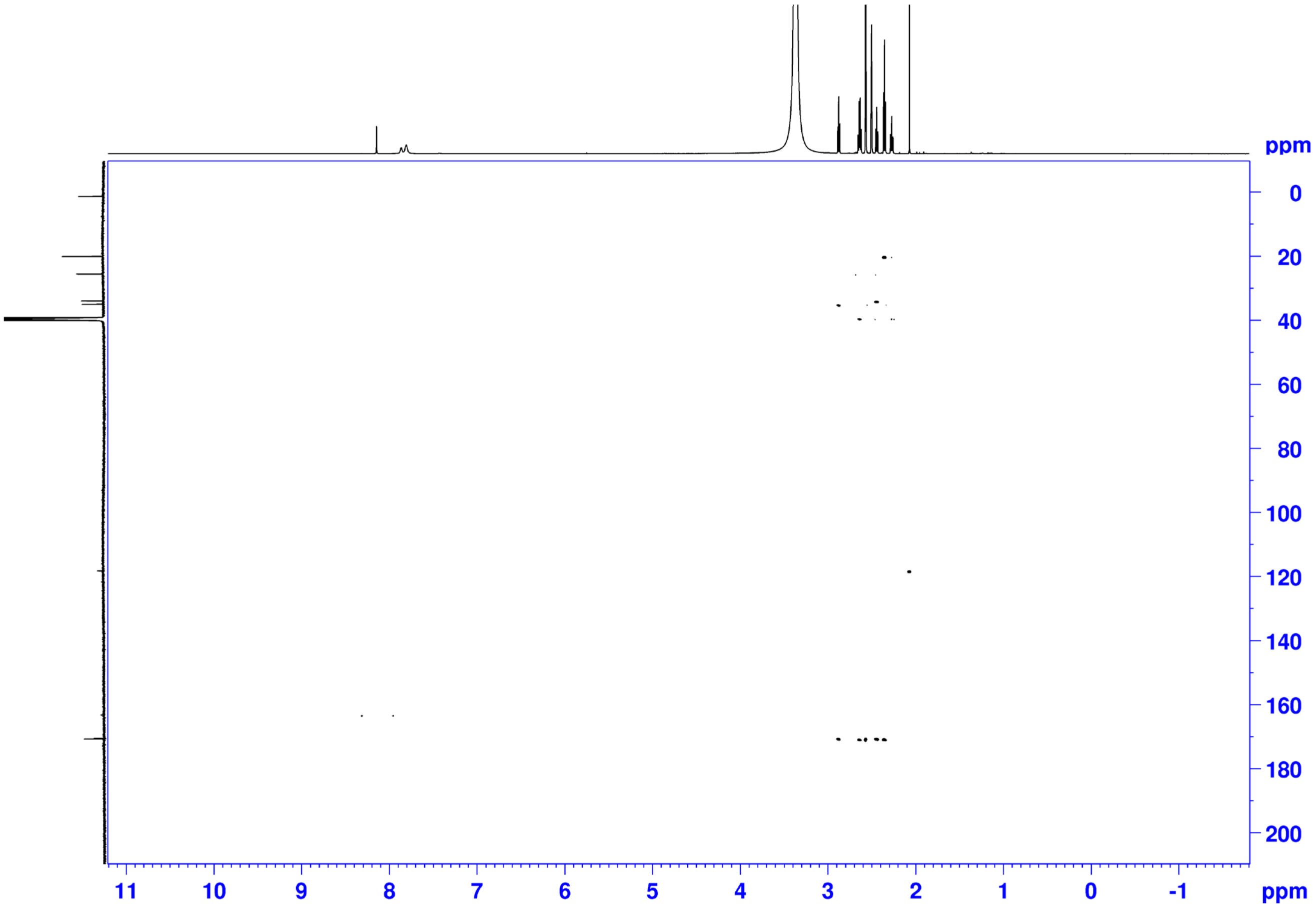

Fig. S11. ^{1}H-^{13}C HMBC NMR (600 MHz, DMSO-d$_6$) spectrum of **3a** and **3**

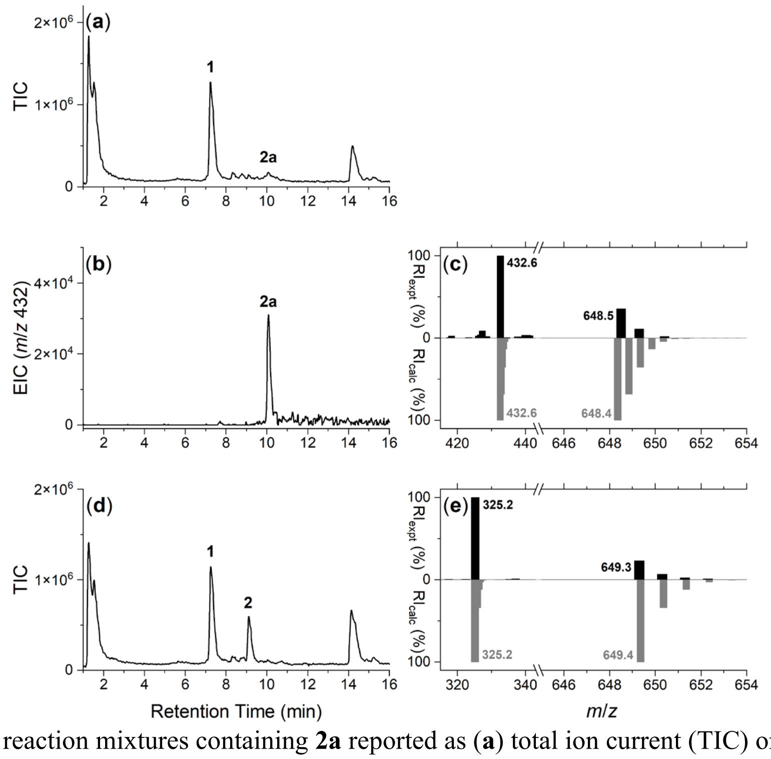

Fig. S12. LC-MS traces from reaction mixtures containing **2a** reported as (**a**) total ion current (TIC) or (**b**) EIC 432 corresponding with the [M+3H]$^{3+}$ adduct, with experimental (black) MS signals from the peak at (**c**) 10.3 min, with calculated signals ([M+2H]$^{2+}$, [M+3H]$^{3+}$ adducts) in grey. LC-MS trace from reaction mixtures of **2a** and TCEP containing **2** reported as (**d**) total ion current (TIC), with experimental (black) MS signals from the peak at (**e**) 9.1 min, with calculated signals ([M+H]$^+$, [M+2H]$^{2+}$ adducts) in grey.

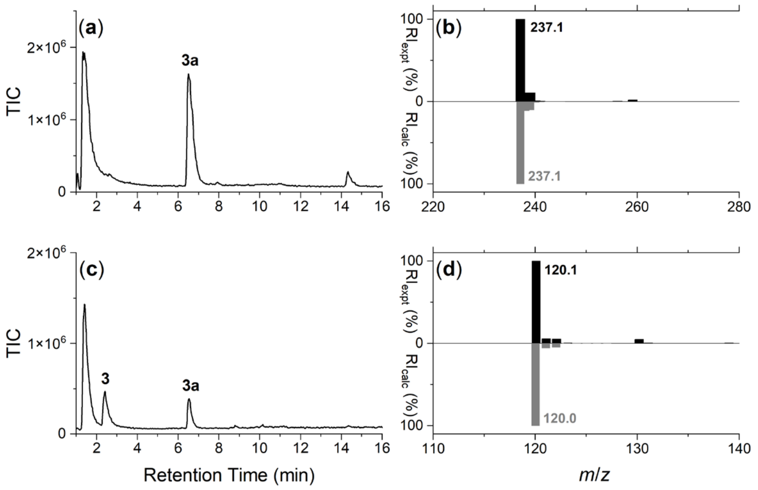

Fig. S13. LC-MS trace from reaction mixtures containing **3a** reported as (**a**) TIC, with experimental (black) MS signals from the peak at (**b**) 6.5 min, with calculated signals ($[M+H]^+$ adduct) in grey. LC-MS trace from reaction mixtures of **3a** and TCEP containing **3** reported as (**c**) TIC, with experimental (black) MS signals from the peak at (**d**) 2.4 min, with calculated signals ($[M+H]^+$ adduct) in grey.

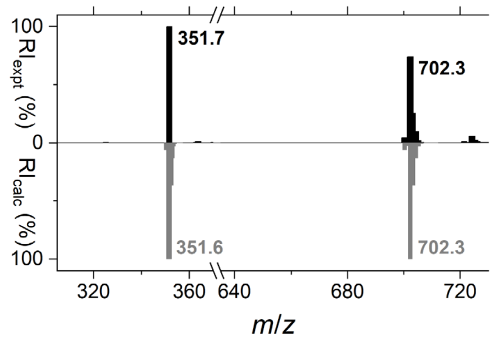

Fig. S14. Experimental (black) MS signals from a solution of Fe(III)-**2** with calculated signals ($[M+H]^+$, $[M+2H]^{2+}$ adducts ($M = C_{28}H_{49}FeN_6O_9S$)) in grey.

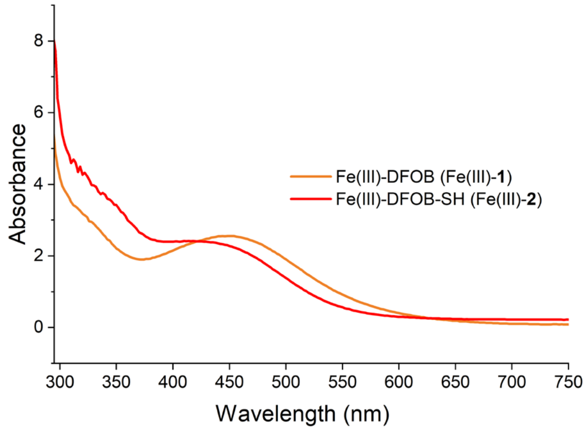

Fig. S15. Electronic spectra from Fe(III)-DFOB (Fe(III)-**1**) (orange) and Fe(III)-DFOB-SH (Fe(III)-**2**) (red).

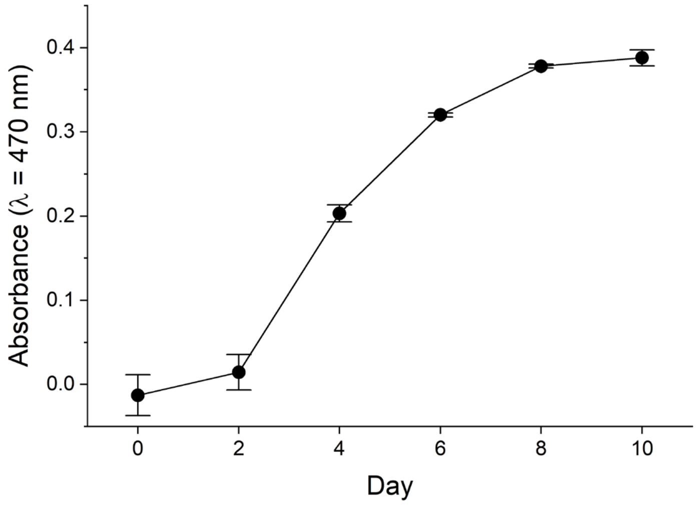

Fig. S16. Absorbance (λ = 470 nm) from culture medium inoculated with *S. pilosus* sub-sampled at 2-d intervals over 0-10 d, with aliquots mixed with Fe(III) prior to analysis. Error bars represent the range for two replicate measurements.

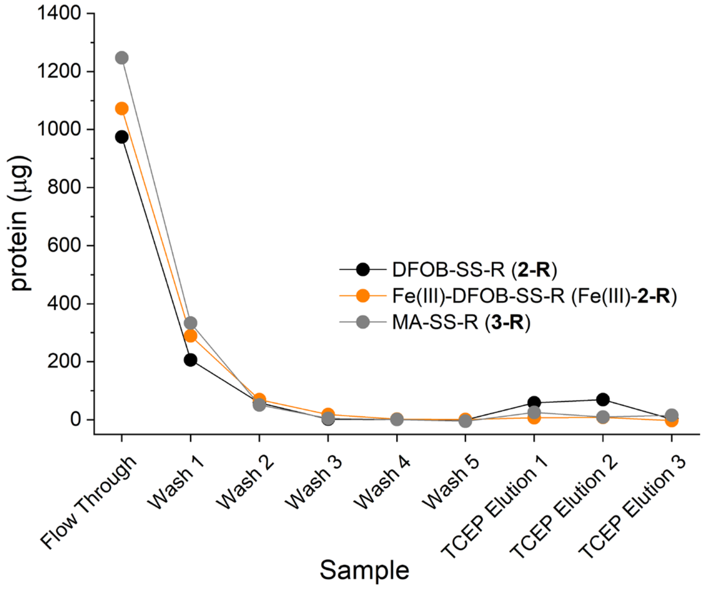

Fig. S17. Protein content (μg) as determined using the Bradford assay in fractions collected from the pulldown procedures using **2-R** (black), Fe(III)-**2-R** (orange) or **R-3** (grey) resins.

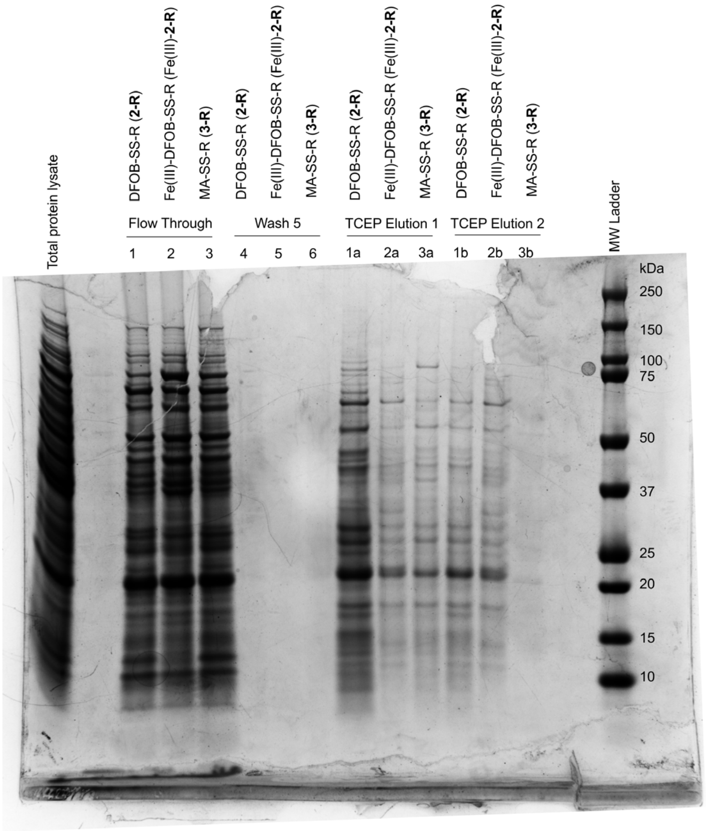

Fig. S18. SDS PAGE gel from fractions collected from the pulldown procedures (referenced to Fig. S2) using **2-R**, Fe(III)-**2-R** or **R-3** resins.

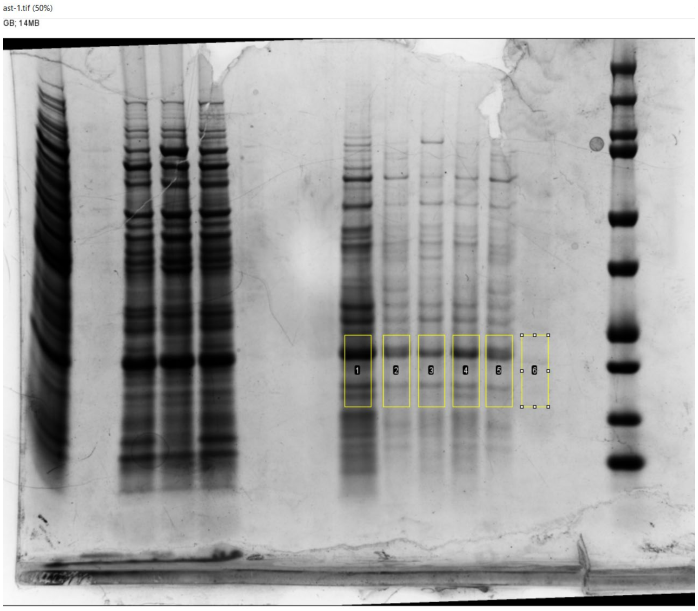

Fig. S19. Regions from the SDS PAGE gel (Fig. S3) sampled for densitometric analysis using ImageJ depcited in the region of interest (Fig. 4b).

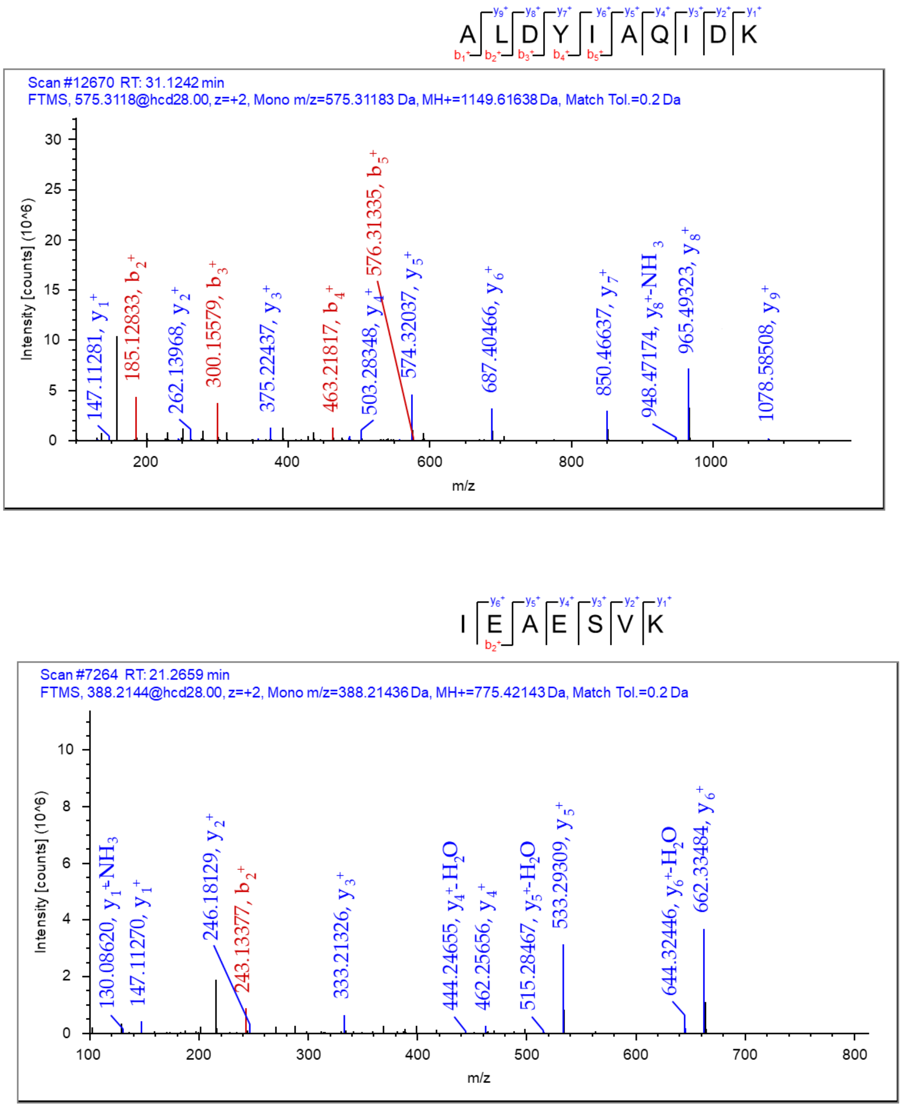

Fig. S20. MS/MS spectrum of peptides (*m/z* 575.3118, where $z = 2$, upper; *m/z* 388.2144, where $z = 2$, lower) from nickel superoxide dismutase (NiSOD; Accession code: P80734) identified from a single band (band *, Fig 2). The *y*- and *b*-ion fragment series are shown.

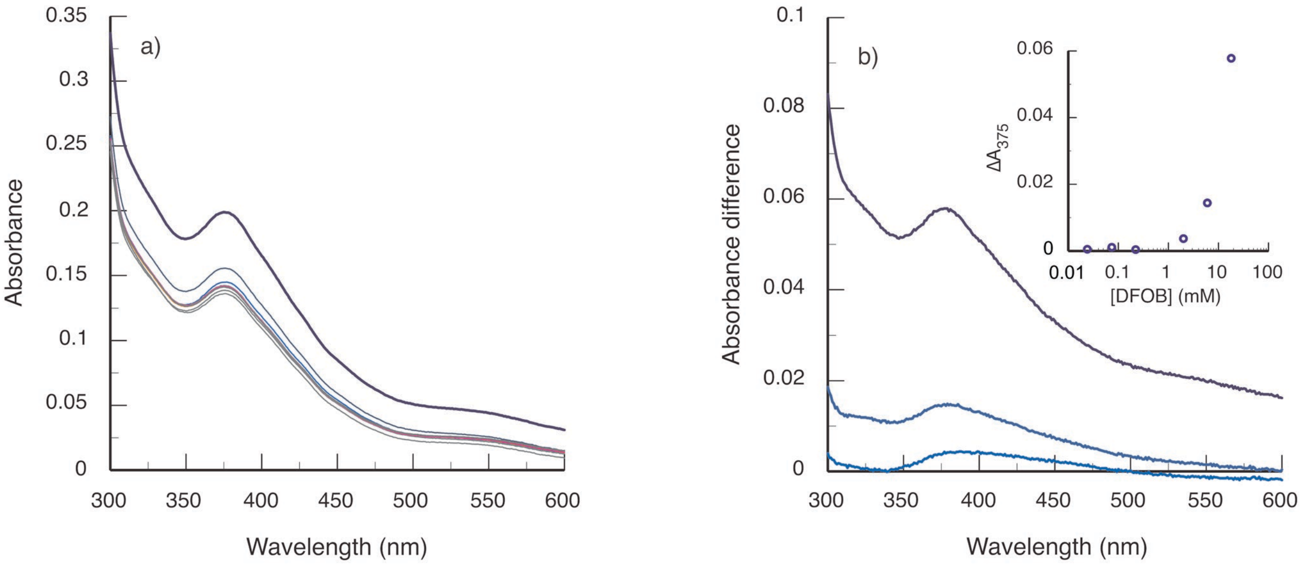

Fig. S21. DFOB-NiSOD interaction probed by UV-visible spectroscopy. a) UV-visible spectra of NiSOD (15 mM) with DFOB from 0 (pink) to 18 mM (blue) from which the corresponding DFOB-buffer only spectrum has been subtracted. b) Difference UV-visible spectra of DFOB-NiSOD from which NiSOD only sample has been subtracted. DFOB concentrations are 3-fold dilutions from 18 mM to 8.2 mM. Inset, A_{375} peak intensity differences from b) plotted as a function of DFOB concentration.

Table 1. MS-MS peptide reads for NiSOD

BEGIN IONS<br>TITLE=Scan 6304 (rt=18.959)<br>[20221020_QEHFX3_<br>MYW_JN_Band10.raw]<br>PEPMASS=388.213 0.00000<br>CHARGE=2+<br>69.070 113070.78<br>70.066 57314.12<br>72.081 65215.11<br>84.081 61593.26<br>86.097 2388678.75<br>87.100 79797.90<br>102.055 82318.42<br>129.102 106377.80<br>130.086 72852.48<br>130.946 32746.88<br>147.113 125300.15<br>187.107 47510.57<br>197.128 38187.02<br>201.086 41416.80<br>215.139 769801.50<br>216.142 48951.51<br>240.098 39341.77<br>243.133 328998.63<br>246.182 98684.84<br>270.107 74227.59<br>288.117 59471.96<br>299.301 78435.02<br>333.212 113645.57<br>333.959 30827.55<br>369.176 70171.40<br>388.214 67722.33<br>389.142 59497.55<br>399.153 34006.35<br>444.242 37699.87<br>462.254 50675.68<br>533.291 769671.00<br>534.297 154212.44<br>644.324 116618.76<br>662.339 443302.31<br>663.336 123110.96<br>END IONS	BEGIN IONS<br>TITLE=Scan 6326 (rt=18.996)<br>[20221020_QEHFX3_<br>MYW_JN_Band10.raw]<br>PEPMASS=388.2131 0.00000<br>CHARGE=2+<br>69.070 156509.92<br>70.066 51865.90<br>71.078 49172.88<br>72.081 53319.93<br>72.098 46445.36<br>74.545 48422.63<br>84.081 79318.81<br>86.097 3709283.75<br>87.100 110858.55<br>92.376 44964.17<br>102.055 106877.96<br>129.102 192783.13<br>147.112 234040.30<br>159.112 52652.85<br>187.144 61963.75<br>197.126 55133.30<br>201.086 54702.51<br>215.139 1145462.25<br>216.142 83943.79<br>243.133 597754.19<br>246.181 109839.15<br>270.110 84679.51<br>288.117 50523.48<br>299.302 97283.64<br>332.684 53582.06<br>333.214 256756.91<br>334.214 50190.27<br>369.176 139410.33<br>382.881 56043.52<br>387.181 54996.00<br>388.214 96311.49<br>399.148 85529.88<br>462.254 94271.88<br>489.953 49567.73<br>491.095 52050.90<br>533.292 1168165.88<br>534.299 218363.61<br>644.322 148288.52<br>662.338 735605.31<br>663.333 139178.88<br>749.418 46862.71<br>END IONS	BEGIN IONS<br>TITLE=Scan 6348 (rt=19.034)<br>[20221020_QEHFX3_<br>MYW_JN_Band10.raw]<br>PEPMASS=388.2131 0.00000<br>CHARGE=2+<br>54.650 42737.64<br>55.668 46187.53<br>69.070 240103.31<br>72.081 79796.02<br>80.196 48846.57<br>84.081 97904.49<br>86.097 4501910.50<br>87.100 128453.38<br>102.055 122185.73<br>104.574 50934.52<br>129.103 192302.03<br>130.086 114640.52<br>147.113 229182.69<br>159.110 60556.72<br>187.107 52975.01<br>187.144 117765.17<br>197.128 84057.30<br>201.086 123006.23<br>215.139 1306913.63<br>216.142 108096.88<br>243.134 558306.94<br>244.133 76182.18<br>246.181 162679.44<br>256.075 50155.88<br>270.107 81372.02<br>288.118 100414.20<br>293.511 52851.66<br>322.719 59904.16<br>333.212 300078.31<br>369.176 141987.75<br>389.141 100507.22<br>426.126 52820.52<br>462.254 92808.23<br>488.234 55099.08<br>498.218 69031.96<br>511.641 57189.44<br>515.276 49948.77<br>533.292 1316319.00<br>534.295 225483.89<br>536.695 58106.47<br>563.266 114483.81<br>587.811 55081.45<br>644.328 184464.27<br>662.340 928857.88<br>663.332 169780.50<br>664.323 72900.63<br>END IONS
BEGIN IONS<br>TITLE=Scan 6370 (rt=19.071)<br>[20221020_QEHFX3_<br>MYW_JN_Band10.raw]<br>PEPMASS=388.2132 0.00000<br>CHARGE=2+<br>69.070 119988.59<br>72.081 48557.94<br>84.082 38716.92<br>85.479 31488.63<br>86.097 2549247.75<br>87.100 65285.60	BEGIN IONS<br>TITLE=Scan 5096 (rt=16.704)<br>[20221020_QEHFX3_<br>MYW_JN_Band10.raw]<br>PEPMASS=488.2402 0.00000<br>CHARGE=2+<br>69.205 55626.95<br>70.066 155795.03<br>74.060 494856.75<br>81.095 44469.85<br>83.061 231848.42<br>84.045 295952.22	BEGIN IONS<br>TITLE=Scan 13490 (rt=31.589)<br>[20221020_QEHFX3_<br>MYW_JN_Band10.raw]<br>PEPMASS=575.3119 0.00000<br>CHARGE=2+<br>86.097 482423.63<br>108.837 14990.44<br>110.071 20513.44<br>116.071 22716.27<br>129.103 32027.21<br>130.087 19476.52

102.055 79467.84<br>103.923 29644.30<br>120.778 29415.96<br>129.102 168642.23<br>130.086 57170.45<br>140.275 31132.38<br>142.123 77680.18<br>147.113 141606.17<br>150.310 32385.82<br>187.145 214100.61<br>197.128 69088.34<br>201.123 54756.86<br>213.653 37296.08<br>215.139 662752.63<br>216.142 74039.91<br>234.144 49604.59<br>240.100 37444.14<br>243.133 315559.78<br>244.133 57841.93<br>270.111 38800.96<br>288.118 39588.45<br>299.315 45162.06<br>333.212 139667.16<br>349.170 53360.94<br>350.122 33442.11<br>369.176 57659.78<br>372.078 35869.17<br>387.188 36721.28<br>388.221 45279.59<br>389.143 75216.73<br>389.198 34385.41<br>399.149 41300.48<br>422.204 40337.33<br>462.254 62866.32<br>464.198 51793.43<br>533.292 682964.50<br>534.295 109524.27<br>563.271 147898.58<br>644.321 69439.06<br>649.997 35391.65<br>662.337 379010.63<br>663.335 80953.59<br>664.319 79424.07<br>780.457 35789.50<br>END IONS	84.081 117942.76<br>84.410 67924.84<br>86.097 58807.56<br>94.051 59434.83<br>113.071 361650.16<br>115.200 54329.41<br>116.071 58894.58<br>129.102 334344.31<br>131.081 1023763.88<br>141.066 226872.34<br>143.081 82655.52<br>146.757 52209.26<br>147.113 207409.56<br>159.076 1226426.13<br>161.092 70665.17<br>166.182 49425.75<br>171.076 256683.23<br>172.638 44581.19<br>189.087 387985.47<br>196.107 92046.52<br>199.071 492432.97<br>203.103 82310.96<br>214.119 91589.47<br>217.082 515120.13<br>224.103 113773.09<br>242.113 486865.59<br>244.093 109932.40<br>268.092 120767.45<br>275.172 58596.20<br>286.102 181622.16<br>301.167 486030.75<br>301.669 128407.15<br>304.113 134325.59<br>332.193 226875.06<br>339.129 146457.28<br>357.139 155675.47<br>357.184 55468.19<br>375.150 138874.44<br>400.201 205437.77<br>433.240 314806.09<br>434.713 116652.67<br>455.226 124953.28<br>473.232 78777.87<br>479.967 57354.98<br>488.222 78428.48<br>488.293 185336.67<br>489.287 66214.88<br>504.279 194516.78<br>583.321 102685.30<br>584.310 83679.38<br>601.332 2987430.50<br>602.334 788090.31<br>603.335 64842.39<br>716.351 622187.81<br>717.358 196116.31<br>756.363 55225.31<br>817.401 390453.88<br>818.402 122038.19<br>END IONS	136.076 74176.71<br>147.113 60373.46<br>157.134 956060.81<br>158.137 64985.84<br>183.150 19733.75<br>185.128 416623.00<br>185.163 17049.74<br>186.132 19289.64<br>200.103 71737.70<br>201.123 24584.46<br>211.144 123430.69<br>217.135 23286.35<br>223.156 28877.70<br>226.119 19754.13<br>229.118 74539.94<br>229.154 42386.48<br>230.115 25174.13<br>244.129 18170.24<br>245.114 64954.48<br>251.104 80262.58<br>251.150 130463.69<br>253.093 27920.43<br>262.139 87347.80<br>277.156 20123.41<br>279.098 67040.62<br>300.155 341355.91<br>301.161 34709.75<br>313.187 62254.55<br>314.170 22786.90<br>326.207 102716.88<br>366.178 50110.60<br>375.222 89123.82<br>392.184 78542.67<br>393.185 17719.63<br>423.652 18312.99<br>428.213 21039.98<br>435.224 62723.43<br>436.229 28224.49<br>459.256 17498.90<br>461.291 35768.66<br>463.222 97766.21<br>464.221 16815.87<br>476.252 18641.80<br>485.273 24689.29<br>486.251 31290.37<br>503.284 45922.27<br>574.321 295147.53<br>575.323 117215.99<br>576.338 67539.71<br>591.282 30270.17<br>661.336 20482.18<br>687.408 195385.28<br>688.403 55180.77<br>704.359 24620.62<br>850.466 148212.42<br>851.468 52859.67<br>852.455 18037.45<br>947.514 17652.53<br>965.494 369017.13<br>966.494 150878.80<br>967.515 32226.92<br>1019.502 19753.15<br>END IONS
BEGIN IONS<br>TITLE=Scan 13512 (rt=31.626) [20221020_QEHFX3_ MYW_JN_Band10.raw]<br>PEPMASS=575.313 0.00000	BEGIN IONS<br>TITLE=Scan 13534 (rt=31.663) [20221020_QEHFX3_ MYW_JN_Band10.raw]<br>PEPMASS=575.3138 0.00000	BEGIN IONS<br>TITLE=Scan 6304 (rt=18.959) [20221020_QEHFX3_ MYW_JN_Band10.raw]<br>PEPMASS=388.213 0.00000

CHARGE=2+<br>86.097 1574392.38<br>136.076 259357.52<br>147.113 155526.20<br>148.418 85656.13<br>154.591 84540.76<br>157.134 3823729.75<br>158.137 207641.73<br>174.015 105551.65<br>185.128 1567201.50<br>200.104 187187.55<br>201.123 98581.87<br>229.120 254594.88<br>251.103 352490.88<br>251.150 141689.70<br>262.139 397590.56<br>277.156 128544.46<br>279.098 217965.52<br>300.155 1192844.00<br>313.186 242382.08<br>326.207 107739.77<br>355.155 109858.63<br>375.222 338526.72<br>392.183 405804.19<br>435.224 243721.17<br>436.228 101981.97<br>439.295 84113.84<br>463.216 375979.94<br>503.284 228402.58<br>574.322 976819.94<br>575.326 241031.27<br>591.271 113893.70<br>687.407 855702.25<br>688.414 195800.13<br>850.469 644313.56<br>851.469 326451.25<br>965.497 1660251.75<br>966.504 598337.81<br>967.489 110984.27<br>END IONS	CHARGE=2+<br>86.092 24758.09<br>86.097 245890.31<br>102.104 15502.34<br>110.071 31890.05<br>116.071 21157.42<br>127.328 14880.92<br>129.103 30643.64<br>136.076 31206.09<br>147.112 22010.22<br>157.134 542455.63<br>158.138 19494.77<br>173.104 15223.32<br>183.150 23561.44<br>185.128 222132.16<br>186.132 18518.58<br>186.414 15192.61<br>200.104 32618.14<br>201.123 21262.67<br>211.144 131296.84<br>223.155 30142.80<br>229.117 42455.99<br>229.154 23264.51<br>244.129 30860.14<br>249.160 22838.42<br>251.103 40520.20<br>251.150 135764.64<br>262.141 31073.69<br>277.162 20185.61<br>279.098 25434.79<br>300.155 194927.13<br>313.187 20554.14<br>323.328 22528.71<br>326.206 136637.45<br>366.177 39708.02<br>375.225 46216.45<br>392.178 51572.66<br>428.211 27114.58<br>435.223 45936.55<br>454.257 16793.72<br>461.284 47636.88<br>463.215 47399.34<br>503.282 45833.10<br>559.869 16685.82<br>574.323 168947.48<br>575.319 72814.45<br>576.338 71960.01<br>687.400 108394.25<br>688.397 23619.89<br>704.359 23717.87<br>850.468 97221.82<br>851.472 31604.88<br>965.494 238569.78<br>966.513 80997.95<br>END IONS	CHARGE=2+<br>69.070 113070.78<br>70.066 57314.12<br>72.081 65215.11<br>84.081 61593.26<br>86.097 2388678.75<br>87.100 79797.90<br>102.055 82318.42<br>129.102 106377.80<br>130.086 72852.48<br>130.946 32746.88<br>147.113 125300.15<br>187.107 47510.57<br>197.128 38187.02<br>201.086 41416.80<br>215.139 769801.50<br>216.142 48951.51<br>240.098 39341.77<br>243.133 328998.63<br>246.182 98684.84<br>270.107 74227.59<br>288.117 59471.96<br>299.301 78435.02<br>333.212 113645.57<br>333.959 30827.55<br>369.176 70171.40<br>388.214 67722.33<br>389.142 59497.55<br>399.153 34006.35<br>444.242 37699.87<br>462.254 50675.68<br>533.291 769671.00<br>534.297 154212.44<br>644.324 116618.76<br>662.339 443302.31<br>663.336 123110.96<br>END IONS
BEGIN IONS<br>TITLE=Scan 6326 (rt=18.996) [20221020_QEHFX3_ MYW_JN_Band10.raw]<br>PEPMASS=388.2131 0.00000<br>CHARGE=2+<br>69.070 156509.92<br>70.066 51865.90<br>71.078 49172.88<br>72.081 53319.93<br>72.098 46445.36<br>74.545 48422.63	BEGIN IONS<br>TITLE=Scan 6348 (rt=19.034) [20221020_QEHFX3_ MYW_JN_Band10.raw]<br>PEPMASS=388.2131 0.00000<br>CHARGE=2+<br>54.650 42737.64<br>55.668 46187.53<br>69.070 240103.31<br>72.081 79796.02<br>80.196 48846.57<br>84.081 97904.49	BEGIN IONS<br>TITLE=Scan 6370 (rt=19.071) [20221020_QEHFX3_ MYW_JN_Band10.raw]<br>PEPMASS=388.2132 0.00000<br>CHARGE=2+<br>69.070 119988.59<br>72.081 48557.94<br>84.082 38716.92<br>85.479 31488.63<br>86.097 2549247.75<br>87.100 65285.60

84.081 79318.81<br>86.097 3709283.75<br>87.100 110858.55<br>92.376 44964.17<br>102.055 106877.96<br>129.102 192783.13<br>147.112 234040.30<br>159.112 52652.85<br>187.144 61963.75<br>197.126 55133.30<br>201.086 54702.51<br>215.139 1145462.25<br>216.142 83943.79<br>243.133 597754.19<br>246.181 109839.15<br>270.110 84679.51<br>288.117 50523.48<br>299.302 97283.64<br>332.684 53582.06<br>333.214 256756.91<br>334.214 50190.27<br>369.176 139410.33<br>382.881 56043.52<br>387.181 54996.00<br>388.214 96311.49<br>399.148 85529.88<br>462.254 94271.88<br>489.953 49567.73<br>491.095 52050.90<br>533.292 1168165.88<br>534.299 218363.61<br>644.322 148288.52<br>662.338 735605.31<br>663.333 139178.88<br>749.418 46862.71<br>END IONS	86.097 4501910.50<br>87.100 128453.38<br>102.055 122185.73<br>104.574 50934.52<br>129.103 192302.03<br>130.086 114640.52<br>147.113 229182.69<br>159.110 60556.72<br>187.107 52975.01<br>187.144 117765.17<br>197.128 84057.30<br>201.086 123006.23<br>215.139 1306913.63<br>216.142 108096.88<br>243.134 558306.94<br>244.133 76182.18<br>246.181 162679.44<br>256.075 50155.88<br>270.107 81372.02<br>288.118 100414.20<br>293.511 52851.66<br>322.719 59904.16<br>333.212 300078.31<br>369.176 141987.75<br>389.141 100507.22<br>426.126 52820.52<br>462.254 92808.23<br>488.234 55099.08<br>498.218 69031.96<br>511.641 57189.44<br>515.276 49948.77<br>533.292 1316319.00<br>534.295 225483.89<br>536.695 58106.47<br>563.266 114483.81<br>587.811 55081.45<br>644.328 184464.27<br>662.340 928857.88<br>663.332 169780.50<br>664.323 72900.63<br>END IONS	102.055 79467.84<br>103.923 29644.30<br>120.778 29415.96<br>129.102 168642.23<br>130.086 57170.45<br>140.275 31132.38<br>142.123 77680.18<br>147.113 141606.17<br>150.310 32385.82<br>187.145 214100.61<br>197.128 69088.34<br>201.123 54756.86<br>213.653 37296.08<br>215.139 662752.63<br>216.142 74039.91<br>234.144 49604.59<br>240.100 37444.14<br>243.133 315559.78<br>244.133 57841.93<br>270.111 38800.96<br>288.118 39588.45<br>299.315 45162.06<br>333.212 139667.16<br>349.170 53360.94<br>350.122 33442.11<br>369.176 57659.78<br>372.078 35869.17<br>387.188 36721.28<br>388.221 45279.59<br>389.143 75216.73<br>389.198 34385.41<br>399.149 41300.48<br>422.204 40337.33<br>462.254 62866.32<br>464.198 51793.43<br>533.292 682964.50<br>534.295 109524.27<br>563.271 147898.58<br>644.321 69439.06<br>649.997 35391.65<br>662.337 379010.63<br>663.335 80953.59<br>664.319 79424.07<br>780.457 35789.50<br>END IONS
BEGIN IONS<br>TITLE=Scan 13490 (rt=31.589)<br>[20221020_QEHFX3_<br>MYW_JN_Band10.raw]<br>PEPMASS=575.3119 0.00000<br>CHARGE=2+<br>86.097 482423.63<br>108.837 14990.44<br>110.071 20513.44<br>116.071 22716.27<br>129.103 32027.21<br>130.087 19476.52<br>136.076 74176.71<br>147.113 60373.46<br>157.134 956060.81<br>158.137 64985.84<br>183.150 19733.75<br>185.128 416623.00<br>185.163 17049.74<br>186.132 19289.64<br>200.103 71737.70<br>201.123 24584.46	BEGIN IONS<br>TITLE=Scan 13512 (rt=31.626)<br>[20221020_QEHFX3_<br>MYW_JN_Band10.raw]<br>PEPMASS=575.313 0.00000<br>CHARGE=2+<br>86.097 1574392.38<br>136.076 259357.52<br>147.113 155526.20<br>148.418 85656.13<br>154.591 84540.76<br>157.134 3823729.75<br>158.137 207641.73<br>174.015 105551.65<br>185.128 1567201.50<br>200.104 187187.55<br>201.123 98581.87<br>229.120 254594.88<br>251.103 352490.88<br>251.150 141689.70<br>262.139 397590.56<br>277.156 128544.46	BEGIN IONS<br>TITLE=Scan 13534 (rt=31.663)<br>[20221020_QEHFX3_<br>MYW_JN_Band10.raw]<br>PEPMASS=575.3138 0.00000<br>CHARGE=2+<br>86.092 24758.09<br>86.097 245890.31<br>102.104 15502.34<br>110.071 31890.05<br>116.071 21157.42<br>127.328 14880.92<br>129.103 30643.64<br>136.076 31206.09<br>147.112 22010.22<br>157.134 542455.63<br>158.138 19494.77<br>173.104 15223.32<br>183.150 23561.44<br>185.128 222132.16<br>186.132 18518.58<br>186.414 15192.61

211.144 123430.69
217.135 23286.35
223.156 28877.70
226.119 19754.13
229.118 74539.94
229.154 42386.48
230.115 25174.13
244.129 18170.24
245.114 64954.48
251.104 80262.58
251.150 130463.69
253.093 27920.43
262.139 87347.80
277.156 20123.41
279.098 67040.62
300.155 341355.91
301.161 34709.75
313.187 62254.55
314.170 22786.90
326.207 102716.88
366.178 50110.60
375.222 89123.82
392.184 78542.67
393.185 17719.63
423.652 18312.99
428.213 21039.98
435.224 62723.43
436.229 28224.49
459.256 17498.90
461.291 35768.66
463.222 97766.21
464.221 16815.87
476.252 18641.80
485.273 24689.29
486.251 31290.37
503.284 45922.27
574.321 295147.53
575.323 117215.99
576.338 67539.71
591.282 30270.17
661.336 20482.18
687.408 195385.28
688.403 55180.77
704.359 24620.62
850.466 148212.42
851.468 52859.67
852.455 18037.45
947.514 17652.53
965.494 369017.13
966.494 150878.80
967.515 32226.92
1019.502 19753.15
END IONS

279.098 217965.52
300.155 1192844.00
313.186 242382.08
326.207 107739.77
355.155 109858.63
375.222 338526.72
392.183 405804.19
435.224 243721.17
436.228 101981.97
439.295 84113.84
463.216 375979.94
503.284 228402.58
574.322 976819.94
575.326 241031.27
591.271 113893.70
687.407 855702.25
688.414 195800.13
850.469 644313.56
851.469 326451.25
965.497 1660251.75
966.504 598337.81
967.489 110984.27
END IONS

200.104 32618.14
201.123 21262.67
211.144 131296.84
223.155 30142.80
229.117 42455.99
229.154 23264.51
244.129 30860.14
249.160 22838.42
251.103 40520.20
251.150 135764.64
262.141 31073.69
277.162 20185.61
279.098 25434.79
300.155 194927.13
313.187 20554.14
323.328 22528.71
326.206 136637.45
366.177 39708.02
375.225 46216.45
392.178 51572.66
428.211 27114.58
435.223 45936.55
454.257 16793.72
461.284 47636.88
463.215 47399.34
503.282 45833.10
559.869 16685.82
574.323 168947.48
575.319 72814.45
576.338 71960.01
687.400 108394.25
688.397 23619.89
704.359 23717.87
850.468 97221.82
851.472 31604.88
965.494 238569.78
966.513 80997.95
END IONS
